# Effectiveness of mobile telemonitoring applications in heart failure patients: systematic review of literature and meta-analysis

**DOI:** 10.1007/s10741-022-10291-1

**Published:** 2023-01-18

**Authors:** Martín Rebolledo Del Toro, Nancy M. Herrera Leaño, Julián E. Barahona-Correa, Oscar M. Muñoz Velandia, Daniel G. Fernández Ávila, Ángel A. García Peña

**Affiliations:** 1 Division of Cardiology, Hospital Universitario San Ignacio, Bogota, Colombia; 2 Department of Internal Medicine, Pontificia Universidad Javeriana, Bogota, Colombia; 3 Department of Internal Medicine, Hospital Universitario San Ignacio, Bogota, Colombia; 4 Colombia GRADE Network, Bogota, Colombia; 5 Division of Rheumatology, Hospital Universitario San Ignacio, Bogota, Colombia

**Keywords:** Heart failure, Telemonitoring, Mobile applications, Smartphones, mHealth, Self-management

## Abstract

Close and frequent follow-up of heart failure (HF) patients improves clinical outcomes. Mobile telemonitoring applications are advantageous alternatives due to their wide availability, portability, low cost, computing power, and interconnectivity. This study aims to evaluate the impact of telemonitoring apps on mortality, hospitalization, and quality of life (QoL) in HF patients. We conducted a registered (PROSPERO CRD42022299516) systematic review of randomized clinical trials (RCTs) evaluating mobile-based telemonitoring strategies in patients with HF, published between January 2000 and December 2021 in 4 databases (PubMed, EMBASE, BVSalud/LILACS, Cochrane Reviews). We assessed the risk of bias using the RoB2 tool. The outcome of interest was the effect on mortality, hospitalization risk, and/or QoL. We performed meta-analysis when appropriate; heterogeneity and risk of publication bias were evaluated. Otherwise, descriptive analyses are offered. We screened 900 references and 19 RCTs were included for review. The risk of bias for mortality and hospitalization was mostly low, whereas for QoL was high. We observed a reduced risk of hospitalization due to HF with the use of mobile-based telemonitoring strategies (RR 0.77 [0.67; 0.89]; I^2^ 7%). Non-statistically significant reduction in mortality risk was observed. The impact on QoL was variable between studies, with different scores and reporting measures used, thus limiting data pooling. The use of mobile-based telemonitoring strategies in patients with HF reduces risk of hospitalization due to HF. As smartphones and wirelessly connected devices are increasingly available, further research on this topic is warranted, particularly in the foundational therapy.

**Supplementary Information:**

The online version contains supplementary material available at 10.1007/s10741-022-10291-1.

## Introduction

Heart failure (HF) is a global health problem that has a negative impact on the quality of life (QoL) of patients [1]. An overall prevalence of 1–2% is estimated, which increases with age, being the most frequent mortality cause in patients older than 65 years [2–4]. Most patients with HF are hospitalized at least once a year [5].

Close and frequent follow-up of these patients by multidisciplinary teams has demonstrated to reduce mortality and hospitalizations due to acute HF [6–8]. However, it is difficult to ensure strict monitoring, so alternative strategies such as telemonitoring are gaining ground [9]. This approach allows to obtain and provide information on patient’s health status though a virtual interface, assist care, reduce the frequency of adverse outcomes, improve QoL, speed up access to healthcare, reduce transportation costs, and reduce face-to-face visits [10, 11].

Telemonitoring strategies have improved medication adherence and re-admission rates [12]. Strategies focusing on treatment optimization and self-care seem to be more successful reducing mortality and hospitalizations due to heart failure, compared to those that aim at early detection and management of acute events, probably due to false alerts [13]. Home-based telemonitoring have proven to be an efficient method of educating and motivating the patients [14]. Smartphone-based apps for telemonitoring in HF are advantageous due to their wide availability, portability, low-cost, computing power, and interconnectivity [15, 16]. A growing number of smartphone-based apps with differential complexities are now available [17–20], with variable feedback strategies, including in some cases 24 h support for emergency event detection and management. However, few studies have evaluated their benefits in clinical outcomes, as shown in previous systematic reviews [16, 21–27].

In this systematic review of RCTs, we evaluated mobile-based telemonitoring strategies in patients with HF, assessing their impact on mortality, hospitalization, and QoL, when compared to standard care.

## Methods

### Protocol and registration

This systematic review followed Cochrane methodology [28]. Protocol was approved by the institutional committee (approval code: 005–2022) and registered in the International Prospective Register of Systematic Reviews (PROSPERO), #CRD42018107855. This report is based on the Preferred Reporting Items for Systematic Reviews and Meta-Analyses (PRISMA) statement [29].

### Eligibility criteria

We included randomized controlled trials (RCTs) evaluating adults (> 18 years old) with HF and comparing telemonitoring strategies using mobile applications with usual care, published between 2000 and 2021. A clear HF definition had to be defined (universal definition [30] or an explicit definition from a national or international guideline). We defined telemonitoring mobile application as a tool that should (1) register at least one relevant clinical variable for follow-up (i.e., symptoms, weight, heart rate, blood pressure); (2) offer an interface using any kind of mobile device; and (3) ask the patient to register clinical variables during follow-up. Studies should provide detailed description of clinical decisions derived from registered information (i.e., feedback), and measure at least one effectiveness outcome (mortality, hospitalization, or impact on QoL). For QoL, we included studies reporting any of the following: EQ-5D-5L [31], SF-36 [32], KCCQ [33], and MLHFQ [34]. We excluded non-randomized studies, reviews, abstracts, letters to the editor, case reports, case series, before and after studies, studies with follow-up of less than a month, studies focusing on multiple diseases, and studies using implantable devices or invasive monitoring.

### Search strategy and information sources

A comprehensive literature search was conducted (full search strategy and terms described in Supplemental Appendix). Electronic databases, including PubMed (MEDLINE), EMBASE (Elsevier), BVSalud (LILACS), and Cochrane Reviews from January 1st, 2000, through December 31st, 2021, were searched. We included studies in English and Spanish. Terms used were “heart failure”, “Smartphone”, “telemedicine”, “mobile applications”, “mHealth”, plus filter “randomized controlled trial”, their synonyms and combinations using Boolean terms. We further searched for useful articles using a “snowball strategy” by reviewing references of included articles and searching grey literature. All duplicates and overlapping results were identified and removed in title screening phase.

### Study selection

Study selection was performed by two independent researchers (MRdT, NHL, or JBC) using online application Abstrackr [35]. We reviewed full texts of relevant citations and further screened for eligibility. Disagreements between individual judgments were resolved by consensus or with a third evaluator (OMM), based on recommendations of the Cochrane Handbook for Systematic Reviews [28] and PRISMA statement checklist [29].

### Data collection process

Data was collected in standardized electronic form including study design, inclusion criteria, participant demographics and baseline characteristics (i.e., age, gender, basal functional class according to New York Heart Association classification [36], HF etiology, Left Ventricular Ejection Fraction [LVEF]), HF definition, telemonitoring software type, retrieved variable type, input methodology by patient, output variables for patient and physician, feedback availability, and follow-up time. Outcomes registered were all-cause mortality, mortality due to HF, all-cause or due to HF hospitalizations, and QoL. We did not adjust units for analysis. Data from included studies was collected by two investigators (MRdT, NHL, or JBC). Disagreements were resolved by consensus or with a third evaluator (OMM).

### Assessment of risk of bias in included studies

Two reviewers (MRdT, NHL, or JBC) independently assessed all documents using RoB2 tool. An experienced third reviewer (OMM, AG, or DF) resolved disagreements between individual judgments. All studies were ranked in five different domains yielding results of low risk of bias, some concerns of bias, or high risk of bias. Risk of bias was determined by outcome. Mortality and hospitalization were not likely to be influenced by blinding, whereas measurement of QoL, despite being performed using standardized tools, relies on patients’ subjectivity. Evaluation of evidence certainty for each outcome was performed using GRADE tool [37].

### Data synthesis and analysis

Data synthesis was performed for each evaluated outcome. We reported quantitative variables as median and interquartile range, and dichotomic variables as proportions. If sufficient information was available, we calculated relative risks for all-cause or HF-specific mortality, hospitalization outcomes, and QoL using a random effects model for meta-analysis. We performed subgroup analyses for follow-up time (< 1-year vs. > 1-year), patient feedback (immediate vs delayed), and software type. Data analysis was performed using RevMan 5.4. Finally, we generated summary and evaluation tables of retrieved evidence, including certainty of evidence for each outcome, using GRADEpro Tool.

## Results

### Study selection and characteristics

**Fig. 1:**
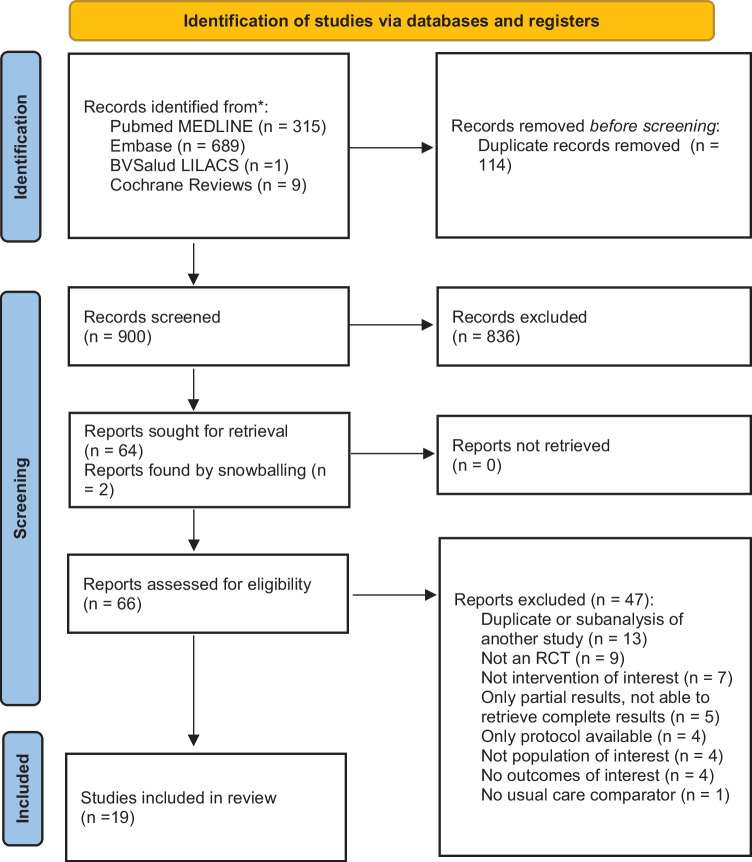
PRISMA

We found 900 references, 66 were reviewed in full text and 19 were finally included in the analysis [22, 25, 38–59]. Selection process is described in Fig. 1. Patient characteristics for each study are presented in Table 1. All included studies were published in English. Most (68%) included less than 100 patients per arm. Mean age was between 48 and 80 years old, with higher proportion of men. Twelve (63%) studies reported HF etiology, ischemic being the most frequent. Fourteen (74%) studies reported mean LVEF: 85% of studies included patients with reduced ejection fraction heart failure. Eleven studies (57%) reported mortality, 13 (68%) hospitalization, and 11 (57%) evaluated QoL. Most studies (63%, *n* = 12) had patient follow-up of less than a year.

**Table 1:** Description of the studies

Study	Intervention/Comparator	Patients, n	Age in years, Mean (SD or IQR)	Men, n (%)	NYHA, n (%)	LVEF, Median (SD or IQR)	Main etiology of HF, n (%)	Follow-up time to outcome in months, median	Main inclusion criteria Definition HF Recent Hx others			Outcomes
1. Scherr et al. [22]	I: home-based TM with MPh (MOBITEL) C: UC	I: 54 C:54	I: 65 (62–72) C:67 (61–72)	I: 40 (74) C: 39 (72)	I: II 7 (13), III 33 (61), IV 14 (26) C: II 7(13), III 37 (68.5), IV 10(18.5)	I: 25 (20–38) C: 29 (21–36)	I: HT 29 (54) C: HT (24 (44)	6	ESC 2005 Guideline OMT according to ESC 2005 guidelines (ACEI/ARA-II, BB and diuretic)	Decompensated HF with Hx > 24 h in the last 4 weeks	> 18 and < 80 years	CV Mort HHF # Days for HHF
2. Vuorinen et al. [38]	I: TM with MPh App C: UC HF Clinic, edu, Self-care, self-monitoring suggestions Telephone follow-up for edu and self-care	I: 47 C:47	I: 58.3 (11.6) C: 57.9 (11.9)	I: 39 (83) C:39 (83)	I: II 19 (40), III 27 (58), IV 1 (2) C: II 17 (36), III 28 (60), IV 2 (4)	I: 27.3 (4.9) C: 28.6 (5)	NR	6	Systolic heart failure, NYHA ≥ 2 LVEF ≤ 35%	< 6 mo since last visit	18–90 years	# Days for HHF
3. Kraai et al. [40]	I: DMS guided by ICT with CDMS + TM C: DMS guided by ICT with CDMS - Computerized system for auto support. of optimization of tx. according to physiological values and medical history - edu + counseling	I: 94 C: 83	I: 69 (12) C: 69 (11)	I: 66 (70) C: 62 (75)	I: II 21 (23) III 51 (57) IV 18 (20) C: II 18 (22) III 49 (60) IV 15 (18)	I: 27 (9.9) C: 28 (9)	I: Isq 45 (48) C: 35 (42)	9	HF based on fluid retention sx / sto Diuretic tx requirement Evidence of structural heart disease, LVEF ≤ 45%	Admission to ICU/CCU or cardiology floor or HF outpatient clinic	≥ 18 years	All cause mort HHF All cause Hx Change in HR -QoL (MLHFQ)
4. Hägglund et al. [41]	I: HIS OPTILOGG (tab) C: UC (Not HF Clinic)	I: 32 C: 40	I: 75 (8) C:76 (7)	I: (66) C: (70)	I: II (38) II (62) C: II (18) III (82)	NR	NR	3	ESC 2012 guideline NYHA II-IV Diuretic Tx	Current Hx	Referred to primary care (exclusion of HF Clinic follow-up)	HR-QoL (KKCQ and Swedish version of SF-36)
5. Pekmezaris et al. [42]	I: TM with American TeleCare Life View + weekly televisits C: Comprehensive outpatient management with monthly follow -up, management based on AHA 2013 guidelines	I: 46 C: 58	I: 48.4 (15.2; 19–93) C: 61.1 (15; 26–90)	I: 26 (57) C: 35 (60)	I: II 13 (28) III 33 (72) C: II 18 (31) III 40 (69)	NR	NR	3	Primary Dx of HF NYHA I-III	Recent Hx discharge	≥ 18 years MMSE score ≥ 21	HHF All cause Hx HR-QoL (MLHFQ)
6. Koehler et al. [43]	I: Remote TM with PDA C: UC	I: 354 C: 356	I: 66.9 (10.8) C: 66.9 (10.5)	I: 285 (80.5) C: 292 (82)	I: II 176 (49.7) III 178 (50.3) C: II 180 (50.6) III 176 (49.9)	I: 26.9 (5.7) C: 27 (5.9)	I: Isq 202 (57.1) C: Isq 194 (54.5)	26 (12–28)	Stable ambulatory chronic HF, optimal treatment according to guidelines NYHA II-III LVEF ≤ 35%	Hx in the last 24 months or LVEF < 25%	≥ 18 years	All cause mort CV mort HHF All cause Hx # days for HHF # days all cause Hx HR -QoL (SF 36)
7. Galinier et al. [46]	I: TM + personalized edu (info package + calls every 3 weeks) C: UC	I: 482 C:455	I: 70.0 (12.4) C: 69.7 (12.5)	I: 354 (73.4) C: 323 (71.0)	I: I 29 (6.1) II 210 (44.2) III 182 (38.3) IV 54 (11.4) C: I 32 (7.1) II 196 (43.4) III 185 (40.9) IV 39 (8.6)	I: 39.3 (14.5) C: 38.1 (15.2)	: Isq 232 (48.2) C: Isq 211 (46.5)	18	NR	Hx due to acute HF in ≤ 12 months	≥ 18 years Access to telephone line or GPRS network	All cause mort CV mort All cause Hx HHF HR—QoL (SF-36)
8. Gjeka et al. [47]	I: TM via smartP + VH app + personalized edu C: UC	I: 47 C: 15	I: 68.1 C: 70	I: 23 (48.9) C: 10 (66.7)	NR	NR	NR	1.5	Primary or secondary Dx of HF, Stage C, NYHA III-IV	NR	NR	HHF All cause Hx
9. Pedone et al. [48]	I: Multiparametric TM with smartP + phone support C: UC (edu, monthly follow-up, telephone availability 2 h/day during the week)	I: 47 C: 43	I: 79.9 (6.8) C: 79.7 (7.8)	I: (30.2) C: (46.8)	I: II (31.9) III (57.4) IV (10.6) C: II (32.6) III (55.8) IV (11.6)	I: 44.4 (12.7) C: 48.2 (13.5)	NR	6	Dx based on echocardiography, NT- proBNP	De novo HF in hx or as 1st dx in outpatient clinic	≥ 65 years	All cause mort All cause Hx
10. Sahlin et al. [49]	I: HIS—OPTILOGG (tab) close loop C: UC (HF Clinic, trimestral visits)	I: 58 C: 60	I: 80 (8) C: 77 (11)	I: 39 (67) C: 32 (53)	I: I 6 (11) II 36 (63) III 15 (26) IV 0 (0) C: I 2 (3) II 39 (65) III 19 (32) IV 0 (0)	NR	I: Isq 26 (45) C: HT 27 (45)	8	ESC 2016 guidelines	HHF ≤ 12mo	NR	All cause mort HHF All cause Hx # days for HHF # days all cause Hx
11. Koehler et al. [25]	I: TM Tab Physio-Gate PG 1000 + edu.(interactive and telephone) C: UC according to ESC 2016 guidelines	I: 765 C: 773	I: 70 (11) C: 70 (10)	I: 533 (70) C: 537 (69)	I: I 3 (0) II 400 (52) III 359 (47) IV 3 (0) C: I 8 (1) II 396 (51) III 367 (47) IV 2 (0)	I: 41 (13) C: 41 (13)	I: Isq 301 (39) C: Isq 323 (42)	12	NYHA II-III FEVI ≤ 45% o ≥ 45% with diuretic	HHF ≤ 12mo	NR	All cause mort CV mort HHF # days lost due to hx HR -QoL (MLHFQ)
12. Dang et al. [51]	I: TM through MPh for case assistance C: UC in HF Clinic (monthly contact and resource use questionnaire)	I: 42 C: 19	I: 53 (9.4) C: 60.3 (9)	I: 28 (66.7) C: 11 (57.9)	I: I 19 (45.2) II 16 (38.1) III 7 (16.7) C: I 10 (52.6) II 7 (36.8) III2 (10.5)	NR	NR	3	NR	Ambulatory with Hf dx	≥ 18 years Anticipated survival ≥ 6mo	HR-QoL (MLHFQ, and SF-36)
13. Soran et al. [52]	I: PC-based home DMS (Alere DayLink HFMS) C: UC (Specialized edu for pt and Dr.; telephone follow-up 1 m and 3 m, face-to-face 6 m; Sto and scale self- monitoring)	I: 160 C: 155	I: 76.9 (7.1) C: 76 (6.8)	I: (31.3) C: (39.4)	I: II (57.5) III (42.5) C: II (59.3) III (40.7)	I: 24.3 (8.8) C: 23.8 (8.7)	I: Isq (56.9) C: Isq (53.5)	6	Dx 1st or 2nd HF LVEF ≤ 40% Sto of HF (dyspnea, orthopnea, NPD, fatigue, edema) OMT according to HFSA 2006	Hx ≤ 6 m	≥ 65 years Medicare beneficiary	CV mort HHF # days all cause Hx HR -QoL (KCCQ)
14. Clays et al. [53]	I: Personal Mobile CDMS (HeartMan) C: UC According to guidelines, HF cardiologist and HF nurse	I: 34 C: 22	I: 61.8 (11) C: 65.2 (9.6)	I: 26 (76.5) C: 17 (77.3)	I: II 26 (83.9) III 5 (16.1) C: II 20 (90.9) III2 (9.1)	I: 32.7 (5.9) C: 31.3 (6.9)	I: Isq 19 (55.9) C: Isq 11 (57.9)	6	HF NYHA II-III LVEF ≤ 40%	Outpatient and stable No Hx in ≤ 1mo	≥ 18 years Adequate cognitive fun	HR -QoL (MLHFQ)
15. Dorsch et al. [59]	I: Mobile app (ManageHF4Life) for self-management C: UC Edu, appointment 2 weeks, periodic calls by nurse	I: 42 C: 41	I: 60.2 (9) C: 62 (9)	I: 28 (67) C: 26 (63)	I: I 1 (2) II 10 (24) III 23 (55) IV 8 (19) C: I 0 (0) II 5 (12) III 27 (66) IV 9 (22)	I: 37.2 (20) C: 38.8 (19)	I: Isq 19 (45) C: Isq 29 (71)	3	LVEF ≤ 40% or > 40% + RAE > 40 mm, BNP > 200 pg/mL or NT- proBNP > 800 pg/mL)	Hx or recently discharged due to HF decompensation	≥ 45 years	HR -QoL (MLHFQ)
16. Boyne et al. [54]	I: TM and edu device C: UC (2005 ESC Guidelines)	I: 197 C: 185	I: 71 (11.9) C: 71.9 (10.5)	I: 115 (58) C: 111 (60)	I: II 110 (56) III 79 (40) IV 8 (4) C: II 109 (59) III 74 (40) IV 2 (1)	I: 36 (28–50) C: 35 (26–42)	I: Isq 99 (50.3) C: Isq 91 (49.2)	12	≥ 1 episodic edema requiring diuretics + FEVI ≤ 40% o diastolic disfunction	NR	≥ 18 years Tx by HF cardiologist and nurse	All cause mort HHF All cause Hx # days all cause Hx
17. Kashem et al. [56]	I: TM w-App (InSight Telehealth System) C: UC (Advanced cardiomyopathies and HF program) (Delivery of TM equipment: scale, BPM, pedometer)	I: 24 C: 24	I: 53 (10) C:54 (11)	I: (72) C: (76)	I: II (42) III (58) IV (0) C: II (43) III (52) IV (5)	I: 25 (3) C: 26 (3)	I: Dil. (56) C: Isq (43)	12	AHA 2001 guidelines NYHA II-IV	≥ 1 Hx and ≤ 6 m	Internet access and basic computer skills	Hx all cause # days All cause Hx
18. Wagenaar et al. [57]	I: UC + attention pathway adjusted to e- health (TM with e-Vita interactive platform) C1: UC + Website (Heartfailurematters.org) - Reminders to use it C2: UC (cardiologist + nurse)	I: 150 C1: 150 C2: 150	I: 66.6 (11) C1: 66.7 (10.4) C2: 66.9 (11.6)	I: 113 (75.3) C1: 112 (74.7) C2: 109 (72.7)	I: I 69 (48.9) II 46 (32.6) III 17 (12.1) IV 9 (6.4) C1: I 57 (39.6) II 53 (36.8) III 17 (11.8) IV 17 (11.8) C2: I 57 (39.9) II 55 (38.5) III 24 (16.8) IV7 (4.9)	I: 35.6 (11.2) C1: 35.2 (11.1) C2: 36.2 (10)	NR	12	ESC 2016 guideline ≥ 3mo since Dx	NR	≥ 18 years Able to fill out questionnaires and take BP and weight measurements Internet access	All cause mort HF mort CV mort Hx HF # Days for HHF HR-QoL (MLHFQ)
19. Wita et al. [58]	I: TM with App in tab C: UC (Cardiology Clinic)	I: 28 C: 32	I: 65.1 (11.7) C: 66.9 (9.3)	I: 23 (82.1) C: 24 (75)	NR	I: 26.6 (7) C: 26.1 (6.7)	I: Isq 13 (46.4) C: Isq 16 (50)	24	HF with reduced LVEF, candidates for CRT according to ESC 2013 guidelines	NR	NR	All cause mort HHF

*SD* standard deviation, *IQR* interquartile range, *NYHA* New York Heart Association, *LVEF* left ventricular ejection fraction, *HF* heart failure, *Hx* hospitalizations, *C* comparator, *I* intervention, *Tx* treatment, *TM* telemonitoring, *MPh* mobile phone, *UC* usual care, *HT* hypertensive, *mo* months, *def* definition, *ESC* European Society of Cardiology, *OMT* optimal medical therapy, *ACEi* angiotensin-converting enzyme inhibitor, *ARB*-*II* angiotensin II receptor antagonist, *BB* beta blocker, *wk* weeks, *mort* mortality, *CV* cardiovascular, *HHF* hospitalization for heart failure, *reHx* rehospitalization, *pt* patient, *WHF* worsening heart failure, *NR* not reported, *DMS* disease management system, *ICT* information and communication technology, *CDMS* computerized decision making system, *auto* automated, *edu* education, *Isq* ischemic, *Sx*/*sto* signs and symptoms, *ICU* intensive care unit, *CCU* coronary care unit, *HR*-*QoL* health-related quality of life, *MLHFQ* Minnesota Living with HF Questionnaire, *HIS* home intervention system, *tab* tablet, *KCCQ* Kansas City Cardiomyopathy Questionnaire, *AHA* American Heart Association, *Dx* diagnosis, *EHFScB*-9 European Heart Failure Self-Care Behavior Scale, *SF*-*36* Medical Outcome Study 36-Item Short Form Health Survey, *DHFKS* Dutch Heart Failure Knowledge Scale, *MMSE* Fol-stein Mini-Mental Status Examination, *PHQ*-*4* Patient Health Questionnaire-4, *PDA* personal digital assistant, *GPRS* general packet radio service, *smartP* smartphone, *VH* Veta health, *NT*-*proBNP* N-terminal prohormone of brain natriuretic peptide, *SECD* self-efficacy for managing chronic disease, *HDS* Health Distress Scale, *CP* communication with physicians, *FVN* visual fatigue numeric, *SBVN* shortness of breath visual numeric, *HFSE*-*30* Heart Failure Self-Efficacy Scale-30, *EHFSC* European Heart Failure Self-Care Behavior Scale, *PC* personal computer, *HFMS* heart failure monitoring system, *Dr* doctor, *PND* dyspnea paroxysmal nocturnal, *HFSA* Heart Failure Society of America, *SCHFI* Self-Care of Heart Failure Index, *RAE* right atrial enlargement, *Dil* dilated, *VAS* visual analog scale, *LV GLS* left ventricle global longitudinal strain

Application characteristics are presented in Table 2. Regarding telemonitoring software, most involved preinstalled or web apps through a smartphone (37%, *n* = 7), while two (10%) included web apps not specifically designed for smartphones. Other studies included wireless tablets (21%, *n* = 4) or proprietary devices (31%, *n* = 6).

**Table 2:** Characteristics of the applications

Study	App Name/Device	Own device or downloadable application (OS)	Monitoring equipment delivered	Monitoring data	Data entry method (patient role)	Patient output	Doctor output	FdB Availability	Other fun. and observations
1. Scherr et al. [22]	MOBITEL	MPh w-App (Nokia 3510) IBI	Basic electronic display Auto BPM + HR	Weight BP HR DoM Freq: QD	Man	None	Continuous access to data via secured website Alarm by Email automatically if OOGM set individually or if ∆ > 2 kg TxMod: Yes. Manual, proposed by Dr	Physician could establish MPh contact for confirmation of parameters and TxMod 24 h technical service	Processing and graphic construction of data Data encryption, access restricted to authorized users
2. Vuorinen et al. [38]	App developed by VTT Technical Research Center in Finland	App pre-downloaded in MPh IBI	scale BPM	Weight BP HR Sto Overall condition Freq. ≥ 1 / week	Man	Alarm if OOGM	web access If OOGM, sto or changes➔ nurse contact pt to consult	Immediate to pt through app	NR
3. Kraai et al. [40]	Health- monitor	Interactive monitor Collects data from monitoring devices via bluetooth	scale Auto BPM ECG	Weight BP Freq: QD ECG (every 2 weeks) Sto (Only if OOGM)	Auto Man. Sto	If OOGM ➔cuest de Sto Alarm. hydrosaline restriction If OOGM + Sto present = alert that you will be contacted by nurse	Alerts by the CDMS to optimize treatment according to collected data Alarm through MPh and email if OOGM	Contact by nurse In < 2 h in case of alarm	NR
4. Hägglund et al. [41]	OPTILOGG	wl Tab	wl scale	QD weight Sto (VAS of general condition) every 5 days	Auto Man. Sto	4 views: 1. Summary of weight, dosage, improvement tips 2. Disease info and lifestyle tips 3. Graphic representation of changes in weight, medication and well-being 4. HF clinic contact details and technical support Self-care tips TxMod in case of ∆ > 2 kg in 3 days Alert to consult if weight gain and no response to diuretic	None Optional: Pt provides HIS to appointment with summary	Telephone call by the patient to the HF clinic or technical service	Daily weigh-in reminder Manual search for healthy lifestyle tips
5. Pekmezaris et al. [42]	American TeleCare LifeView _	Computerized monitoring device connected via wl broadband card or telephone	Scale Rest NR	BP SO2 Weight HR Freq. Q.D	Man	NR	Checked every 24 h during the week and every 72 h on weekends If OOGM, nurse notified the treating physician for TxMod or consultation to the ER	Via telephone by nursing in case of OOGM	NR
6. Koehler et al. [43]	NR	PDA with touchscreen, mobile network and bluetooth connection	scale BPM 3-lead ECG *Accelerometer (not all) Emergency response system (Direct communication button with speaker)	BP Weight ECG Sto Walk 6 min (only subgroup that received accelerometer Freq. Q.D	Auto Man. Sto	Health status identified by color code Schedule with measurements	w-App with patient records and graphical interface Alarm according to individual parameters If health deterioration ➔call by treating Dr. In critical cases, emergency assistance	24-h telephone emergency system Medical support 24 h / 7 days	Data encryption
7. Galinier et al. [46]	NR	Device for answering questions of sto	scale	Weight Sto Freq. Q.D	Auto Man. Sto	NR	Visible alarm for nurses who contacted patients and could indicate assistance with a Dr	Contact with nurse on weekdays	Analysis by expert system with generation of alerts and prediction of decompensation
8. Gjeka et al. [47]	VH: Veta Health	App downloaded in smart MPh with bluetooth	Bluetooth BPM Bluetooth pulse oximeter Scale (Not delivered)	Weight HR SO2 Sto Freq. Q.D	Auto Man. for Sto and Weight	Pop-up notifications, emails, symptom questionnaires Medication Reminders View measurement trends and edu content	Web portal with access to all pt data Alarm if OOGM ➔coordinator (not Dr.) contacts pt and defines relevance of medical consultation	Immediate contact with pt if OOGM	Analysis of info for production in actionable format Deterioration risk assessment
9. Pedone et al. [48]	NR	w app smart P Android	Basic BPM Pulse- oximeter	Weight QD BP BID CF BID SO2 TID Sto	Auto Man. for Sto	Alarm for TM Alarm if OOGM	w app daily assessment Alarm If OOGM, ➔contact pt, adherence check, early appointment, emergency room referral	Phone support business hours to report sto or technical help	NR
10. Sahlin et al. [49]	OPTILOGG	Tab. wl	scale	Weight Sto DoM Freq. Q.D	Auto Man. for Sto	Alarm of deterioration for TxMod and contact with Dr	Optional if pt contributes to consultation	Telephone support if deterioration Technical support business hours	Interactive edu
11. Koehler et al. [25]	Physio-Gate PG 1000 Fontane Software	wl Tab with mobile network connection Analysis system for intelligent TM	scale BPM Pulse- oximeter ECG 3 channels	Weight BP HR rhythm analysis SO2 Sto (health status scale 1–5) Freq. Q.D	Auto Man. for Sto	Availability of MPh (Doro) delivered for emergent contact	Access by telemedical staff to the telemedical analysis system Fontane: Direct communication with pt and treating physician, TxMod, coordinate face-to-face visit or hx Access to electronic record by treating physician	Medical support and pt management 24 h / 7d	Algorithm to identify critical or missing values Classification in high /low risk with TM data + MR- proADM values every 3 mo Interactive edu Confidentiality
12. Dang et al. [51]	Model FG 630 (MPh)	Questionnaire via web-browser message in MPh	NR	Weight Sto (9 questions) Freq. Q.D	Man	Reminder to fill out questionnaire Alarm if risk of deterioration ➔contact coordinator	Access via website to data Alarm if risk of deterioration ➔contact pt	Coordinator establishes contact if deterioration risk Monthly telephone contact	NR
13. Soran et al. [52]	Alere DayLink HFMS	Proprietary monitor: Sto monitor system with telephone line connection	Digital scale	Weight Sto Freq. Q.D	Auto Man for Sto	NR	Access to computerized database with graphic trends Alarm if OOGM	Daily review (365d) by nurse If OOGM: Contact pt to verify Contact a Dr. for TxMod, recommend consultation	NR
14. Clays et al. [53]	HeartMan	CDMS app on smartP (Nokia 6 TA_1021)	scale BPM Wrist Sensor (HeartMan BITTIUM) Pill Organizer (PutTwo)	Weight BP HR temperature FR Acceleration Freq. Q.D	Auto	Reminder measurements, medications and appointments Graphic presentation of data Alarm if OOGM to contact Dr	Data and graphics web interface	Technical support business days 9am-4 pm	Edu Exercise schemes and personalized lifestyle recommendations Psychological support (mindfulness, CBT)
15. Dorsch et al. [59]	ManageHF4Life	Self-management app for smartP	scale (Fitbit Charge 2)	Weight Sto Freq. Q.D *TM of other variables, optional	Auto Man for Sto	Color-coded health status indicator (based on weight and sto) with self-management recommendations Measurement Reminder	NR	NR	Edu
16. Boyne et al. [54]	Health Buddy	Own device with display and 4 buttons	NR	Sto	Man	Dialogues of edu, behavior and sto, adaptable to the pt	Care Desktop PC platform Access to answers and risk profiles	Wrong answers ➔immediate correction Sto or high risk ➔contact by nurse	Generation of risk profiles according to responses Take HR and BP during face-to-face meetings
17. Kashem et al. [56]	InSight Telehealth System	w-App	Scale Digital BPM Pedometer	Weight BP HR Steps a day Sto (5 questions) Freq. Q.D	Man	Web access with unique ID Visualization of TM, laboratory and medication data Could send short messages to the Dr	Web access to database of 10–15 patients at a time	Nurse: web message reply in < 1 day Dr.: Could receive standard or individualized messages In an emergency, pt had to call a Dr. / hospital	Encryption of data transfer
18. Wagenaar et al. [57]	e-Vita	w-App for custom TM	Scale BPM	Weight BP HR Freq. Q.D Co-morbidities Medicines Freq. monthly	Man	NR	e-Vita Platform Alarm if OOGM or if no data registration	Nurse: Contact pt if OOGM—> query sto, TxMod, indicate consultation	NR
19. Wita et al. [58]	NR (Developed by Meditel Company in Poland)	App in tab	Scale BPM 3-lead ECG	Weight BP Sto Freq. Q.D ECG every week	Man	NR	Management based on trends of previous week parameters	Possibility of teleconsultation	NR

*app* application, *os* operating system, *FdB* feedback, *fun* functionalities, *w*-*App* web application, *MPh* mobile phone, *IBI* issued bt investigators, *BPM* sphygmomanometer, *auto* automated, *HR* heart rate, *BP* blood pressure, *freq* frequency, *DoM* dosing of medication, *Q*.*D*. once a day, *man* manual, *OOGM* out of goal measurements, *∆* change, *pt* patient, *TxMod* treatment modification, *ECG* electrocardiogram, *Sto* symptoms, *quiz* questionnaire, *Nrs* nursing, *CDMS* computerized decision making system, *HIS* home intervention system, *Tab* tablet, *wl* wireless, *SO*_*2*_ oxygen saturation, *info* information, *VH* Veta health, *smartP* smartphone, *BID* 2 times a day, *TID* 3 times a day, *MR*-*proADM mid* regional pro-adrenomedullin, *msg* message, *HFMS* heart failure monitoring system, *CBT* cognitive behavioral therapy

Most frequently monitored variables were weight (95%, *n* = 18), symptoms (79%, *n* = 15), blood pressure (57%, *n* = 11), and heart rate (42%, *n* = 8). Regarding data entry method, manual input was most frequent (95%, *n* = 18), although ten of the studied strategies (53%) reported both, manual and automatic interface using wirelessly connected external equipment (e.g., scales, blood pressure monitors, etc.). Most (*n* = 18) had a feedback plan; however, only 3 (16%) explicitly stated having immediate (< 2 h) support. Only 4 (21%) declared having 24 h availability.

### Risk of bias assessment

RoB2 domain scores for each included study are shown in Supplemental Fig. 1. Only two (10%) RCTs were ranked as low risk of bias [49, 54, 55], whereas twelve (63%) presented at least some concerns of bias with regard to outcomes such as mortality and/or hospitalization.

### All-cause and HF-specific mortality

**Fig. 2:**
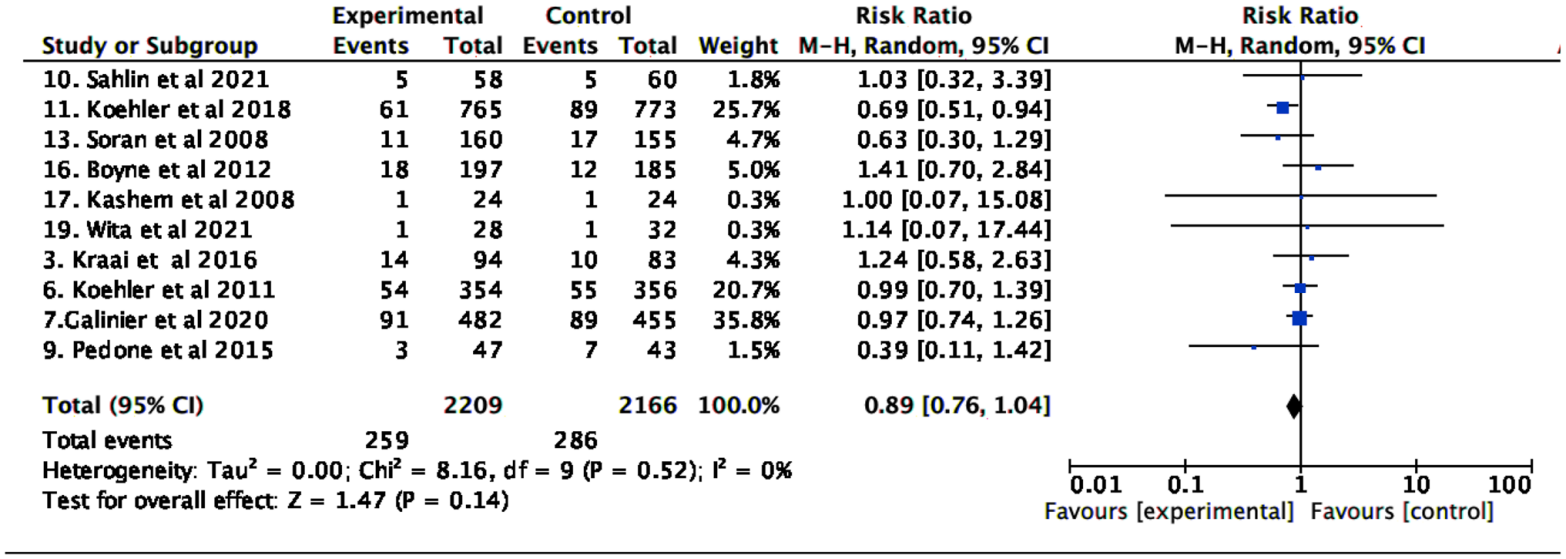
All-cause mortality

**Fig. 3:**
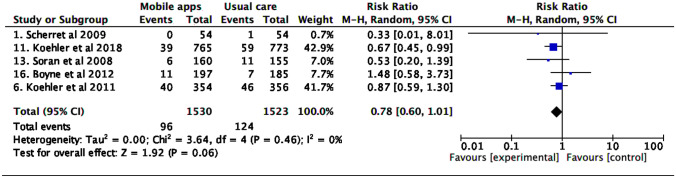
Cardiovascular mortality

In the global analysis, no differences were found in the risk of all-cause and cardiovascular mortality (Figs. 2 and 3).

### All-cause and HF-specific hospitalization rate

**Fig. 4:**
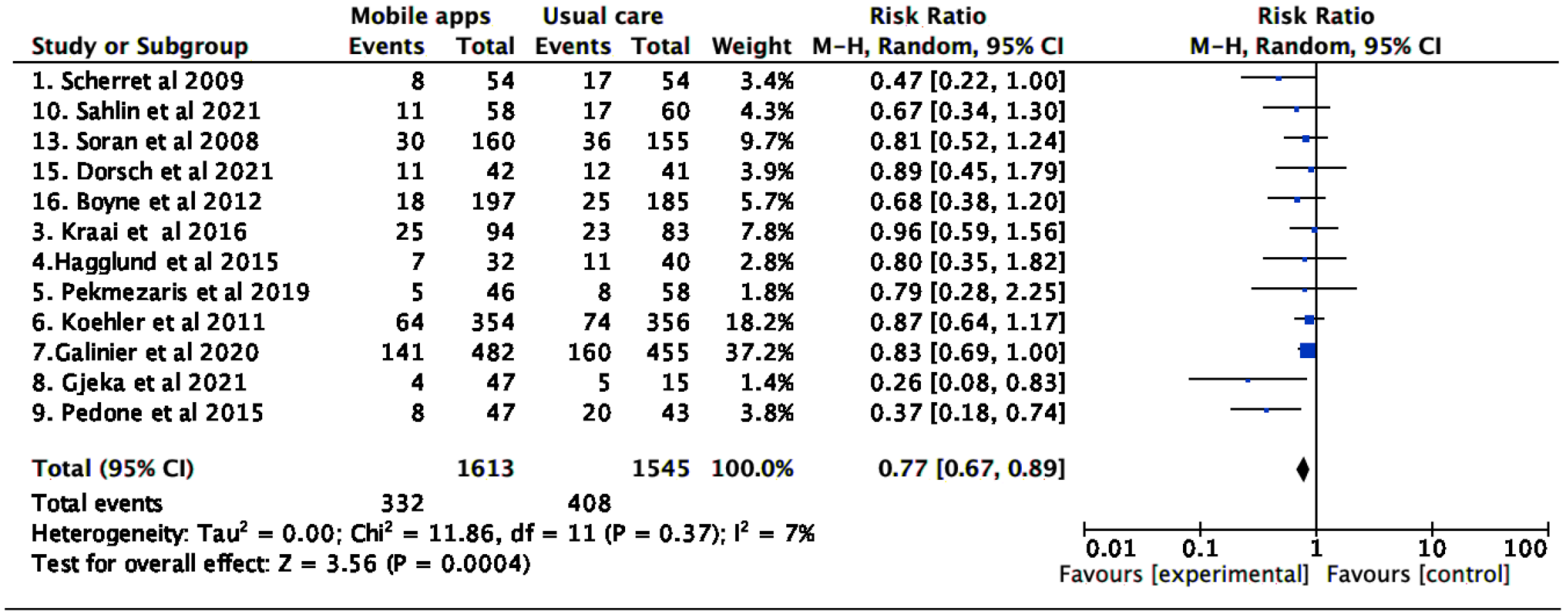
Heart failure hospitalization

**Fig. 5:**
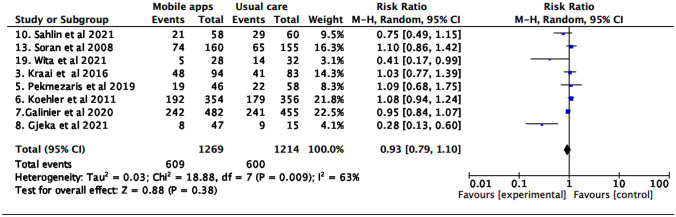
All-cause hospitalization

Tele monitoring strategies using mobile applications reduced HF hospitalization (RR 0.77 [0.67; 0.89], I^2^ 7%). No differences were found in the risk of all-cause hospitalization (Figs. 4 and 5).

### Quality of life

Several scores to evaluate QoL were used in included studies (*n* = 11) (Table 3). Most frequently used tools were MLHQ [34] (64%, *n* = 7), SF-36 [32] (18%, *n* = 2), KCCQ [33] (9%, *n* = 1), and EQ-5D [31] (9%, *n* = 1). Due to heterogeneity in effect measurement report, pooled analysis was not possible. No improvement in QoL was observed in studies using MLHQ [25, 40, 42, 53, 57, 59, 60] or EQ-5D [54], whereas studies applying SF-36 [43, 46] and KCCQ [41] reported statistically significant improvement. Noteworthy, one study was not included as it only reported QoL previous to intervention [52]; further, two studies [60, 61] measured QoL using two different tools, but only presented complete data for one tool.

**Table 3:** General characteristics of studies evaluating Quality of Life

Trial	Score used	Follow-up, months	Group	Number of patients	Initial score, media (SD)	Final score, media (SD)	Change, media (SD)	*p*
3. Kraai et al. [40]	MLHFQ	9	TM	60	47.2 (20.6)		− 13.97 (22.311)	0.63
			Usual care	58	46.3 (25.1)		− 14.63 (25.14)	
5. Pekmezaris et al. [42]	MLHFQ	3	TM	46	62.7	36.3		0.50
			Usual care	58	59.9	27.8		
11. Koehler et al. [25]	MLHFQ	12	TM	649			− 3.08	0.26
			Usual care	624			− 1.98	
12. Dang et al. [51]	MLHFQ	3	TM	36	46.7 (25.6)	42.8 (27)	− 3.94 (26.2)	0.43
			Usual care	16	44.1 (24.4)	44.8 (26.4)	0.75 (16)	
14. Clays et al. [53]	MLHFQ	6	CDMS	34	32.1 (22.9)		− 1 (14.4)	0.50
			Usual care	22	30 (13.5)		− 1.7 (13.8)	
15. Dorsch et al. [59]	MLHFQ	3	App	42	55.6 (3.5)	44.2 (4)		0.78
			Usual care	41	59.2 (3.4)	45.9 (4)		
18. Wagenaar et al. [57]	MLHFQ	12	Website	150	24 (31)	28.3		
			E-Health	150	23 (27.8)	25.5		
			Usual care	150	23 (32.5)	26.5		
6. Koehler et al. [43]	SF-36*	26	TM with PDA	354	54.3 (1.2)	53.8 (1.4)	1.7	0.01
			Usual care	356	49.9(1.2)	51.7 (1.4)	0.3	
7. Galinier et al. [46]	SF-36**	18	TM	482	37.4 (18.8)	11.1 (21.8)		0.03
			Usual care	455	39 (19.2)	7.3(21.7)		
16. Boyne et al. [54]	EQ-5D	12	Device with TM	179	0.64 (0.3)	0.65 (0.2)	0.01	0.83
			Usual care	173	0.61 (0.3)	0.63 (0.3)	0.02	
4. Hägglund et al. [41]	KCCQ’s	3	Wireless tablet	32	50	65.1		< 0.05
			Usual care	40	42.7	52.1		

*MLHFQ* SF-36 short Form-36, *KCCQ* Kansas City Cardiomyopathy Questionnaire, *SD* standard deviation, *TM* telemonitoring, *CDMS* computerized decision making system, *app* application, *PDA* personal digital assistant

*SF 36 Physical component

**SF 36 Vitality score

### Subgroup analysis

**Fig. 6:**
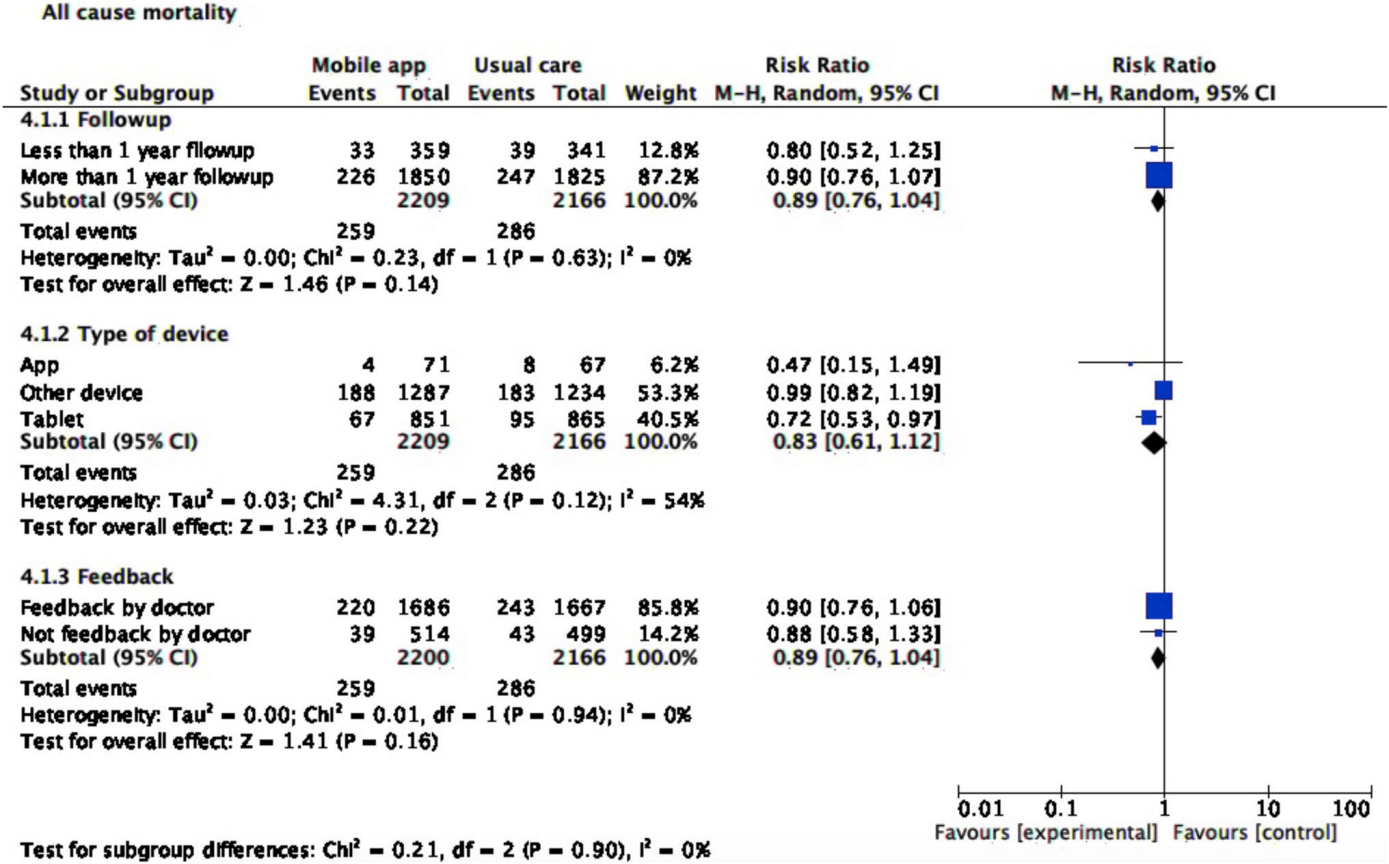
All-cause mortality subgroup analysis

**Fig. 7:**
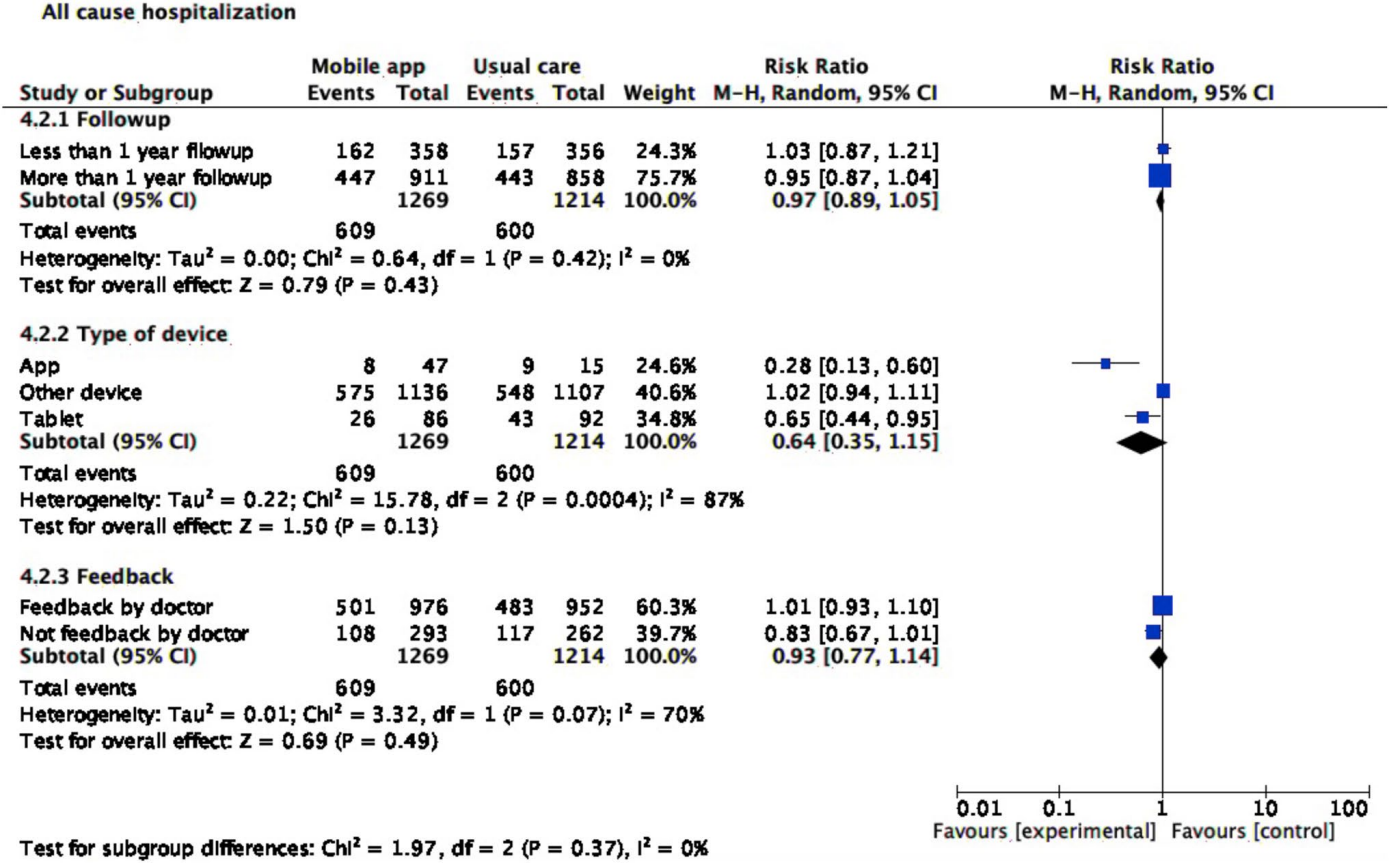
All-cause hospitalization subgroup analysis

**Fig. 8:**
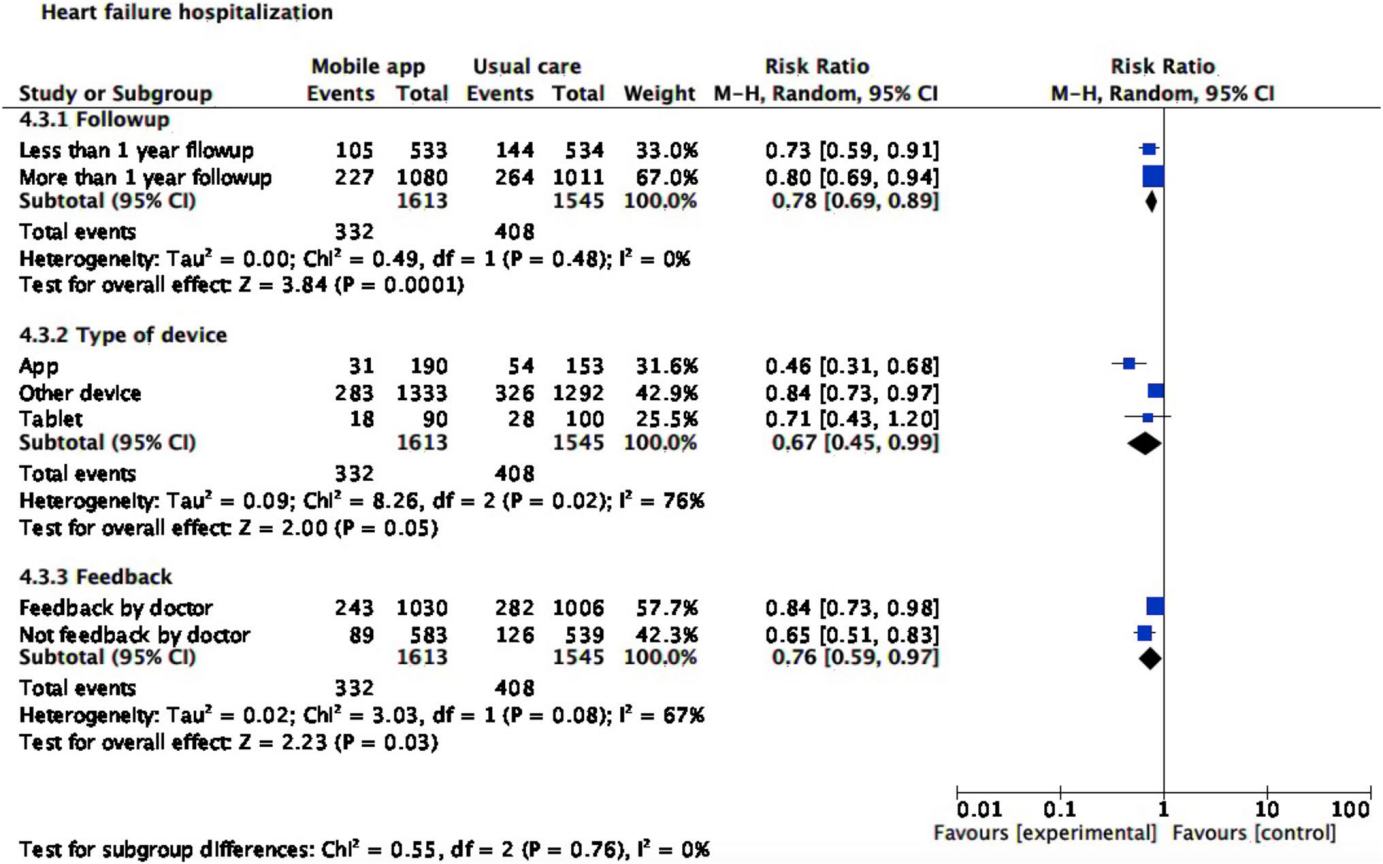
Heart failure hospitalization subgroup analysis

For subgroup analyses (Figs. 6, 7, and 8), we stratified studies by follow-up length (less or more than a year), device type (Smartphone application, tablet, or other device), and feedback (by physician or not). With regard to mortality, tablet use was associated with lower all-cause mortality risk (RR 0.72, CI 95% 0.53, 0.97). Smartphone application or another device as monitoring strategy was associated with lower risk of both all-cause (RR 0.28, CI 95% 0.13,0.60 for smartphone application; RR 0.65, CI 95% 0.44,0.95 for tablet) and cardiovascular hospitalization (RR 0.46, CI 95% 0.31,0.68 for smartphone application; RR 0.84, CI 95% 0.73,0.97 for another device). Meanwhile, cardiovascular hospitalization was reduced in the intervention group, regardless of follow-up length (RR 0.78, CI 95% 0.69, 0.89) and feedback type (RR 0.76, CI 95% 0.59, 0.97).

### GRADE

Supplementary Table 1 describes the summary of findings and evidence certainty evaluation. Certainty of evidence for both all-cause mortality and cardiovascular hospitalization was moderate, whereas for cardiovascular mortality and all-cause hospitalization was low. Certainty of evidence for QoL differed between applied tool, with high certainty level for EQ-5D (only one study), moderate for SF-36, and low for MLHFQ and KCCQ’s.

## Discussion

This systematic review evaluated impact of telemonitoring strategies using mobile applications for patients with HF. We found their use reduces HF hospitalization risk (RR 0.77, [0.67; 0.89]) with low heterogeneity. No significant differences were found for all-cause and cardiovascular mortality, and all-cause hospitalization. Regarding QoL, several scores have been evaluated with different reporting strategies limiting pooled analysis; their impact was divergent between studies. Most studies presented at least some concerns of bias.

Most strategies that reduce hospitalization risk in patients with HF rely on pharmacologic approach [1, 62]. Nonetheless, adherence to therapy and guidelines’ recommendations are suboptimal [63, 64]. As illustrated by our results, mobile-based software for telemonitoring patients with HF may positively impact this risk. Previous meta-analyses [65, 66] including studies of home-based monitoring for patients with HF, showed these strategies reduce re-admission events, due to earlier detection of decompensation and therapeutic intervention; in addition, it promotes treatment adherence. In addition, telemonitoring strategies can reduce the frequency of unnecessary hospital visits, which has been of great importance during Covid-19 pandemic [11].

Smartphone-based apps for telemonitoring in HF are beneficial due to their wide opportunity, cheapness, and computational power [15, 16]. Current evidence suggests positive impact on treatment adherence and reduction in HF hospitalization [12, 16, 22–24]. We recently published a pilot study in 20 patients followed for 6 months at our institution using real-time telemonitoring smartphone App (“ControlVit”), in which we found that 91% of patients who used the App did not present any hospitalization event [12].

In 2016, Cajita MI et al. published a systematic literature review exploring impact of mobile phone-based interventions in patients with HF, which included 9 studies (5 were RCTs), reporting inconclusive findings regarding mortality, readmissions, hospitalization duration, QoL, and self-care [26]. The readmission risk assessment included only three studies and less than half of the patients included in the present review, possibly explaining differences with our results. Further, a more recent pooled analysis by Son YJ et al. reported mobile-based interventions had significant impact on in-hospital management duration. Nonetheless, authors did not find differences in all-cause mortality, readmissions, emergency department visits, or QoL 27. In contrast to our study, the most frequent intervention was voice-call feedback, in which an interface for telemonitoring interaction was lacking; thus, evaluated interventions were rather different.

Noteworthy, our results did not show a definite impact on mortality. Few interventions have demonstrated to reduce mortality in this patient group. Out of 19 included studies, we found that only one RCT showed reduction in mortality. Koehler et al. [25] evaluated telemonitoring using a wirelessly connected tablet, in which variables such as symptoms, vital signs and heart rate were retrieved. We hypothesize the positive impact was because feedback was available 24 h/7 days. Further, this strategy was based on an algorithm identifying critical values and able to classify patients in different risk strata [25]. New studies are needed to assess whether the potential benefits of closer feedback and automated algorithms are consistent.

Regarding evidence quality, we found most RCTs presented at least some concerns of bias. This phenomenon may be explained by a couple of reasons. As measure of QoL relies on patient’s subjectivity, it yields a high-risk of bias in the evaluation process. This limitation is less important for main outcomes such as mortality and readmission. Most studies (4/5) were considered as low risk of bias RCTs with regard to those outcomes. Remaining studies had mainly limitations on their randomization, as information concerning concealing was lacking, or due to baseline differences between study arms.

We acknowledge some drawbacks of our study. First, most studies were performed before widespread sacubitril/valsartan and iSGLT2 use, which has been one the most important advances in HF management, as it reduces mortality and hospitalization risk across the whole heart failure spectrum [1, 62, 67]. We were unable to ascertain pharmacologic treatment and patient adherence. Thus, our results may differ during foundation therapy era, as several novel agents have become first-line therapy in HF management armamentarium [1, 62]. Nonetheless, smartphone-based telemonitoring implementation is a low-cost and widely available strategy warranting further exploration in high-quality RCTs. Second, the fact we included different strategies for telemonitoring, using not only smartphone-based apps, but external devices and web-based forms, may be considered a limitation for comparisons. We recognize the heterogeneity among included mHealth interventions. However, our telemonitoring definition finds common basic characteristics, illustrating a process in which there is (1) patient input, (2) data processing, and (3) output allowing both feedback and decision-making. As smartphone availability is increasing and access to wirelessly connected external devices (e.g., smartwatch, scales) is spreading, impact of such devices on real-time data input and decision-making should be explored. For instance, data from Apple Watch® has been shown to be useful in arrhythmia detection [68]. Seeking to minimize this possible bias, we performed a subgroup analysis to assess possible heterogeneity secondary to device type without significant differences. Third, interpretation and data pooling for QoL was limited due to the use of different tools. As interest on impact of patient-reported outcomes is increasing, a call is warranted to establish a preferred tool and to standardize reporting of this outcome. This will allow data pooling in meta-analysis. In addition, novel approaches for composite outcomes analysis, such as win ratio [69], allow inclusion of QoL scores in RCTs. This approach should be considered in data analysis of RCTs evaluating telemonitoring. Fourth, follow-up times were uneven between studies, thus limiting data interpretation. Future studies on smartphone app telemonitoring should consider a minimum and ideally longer follow-up time. We acknowledge that differences in inclusion criteria and HF definition across studies make it challenging to determine in which HF subpopulations we can expect a positive effect on HF hospitalization. HF definitions have evolved over time, and future RCTs should probably include the recently proposed universal definition [30], allowing a more homogenous set of patients.

## Conclusion

HF is a burdensome entity from an individual and a societal perspective. Despite widespread mobile device availability and its frequent use by patients at-risk or with established HF, mobile-based telemonitoring of HF patients is still a growing area of research. To the best of our knowledge, we offer the most comprehensive and updated systematic review on this topic, demonstrating reduction in HF hospitalization risk in patients using this strategy. Reduction in mortality risk was not statistically significant, warranting further exploration in high-quality RCTs in the foundational therapy era. Future studies on this topic should allow a better assessment of QoL.

## Supplementary Information

Below is the link to the electronic supplementary material.

Supplementary file1 (DOCX 189 KB)

## Data Availability

All data will be available at request to the authors.

## References

[1] McDonagh TA, Metra M, Adamo M (2021). 2021 ESC Guidelines for the diagnosis and treatment of acute and chronic heart failure. Eur Heart J.

[2] Solomon SD, Dobson J, Pocock S (2007). Influence of nonfatal hospitalization for heart failure on subsequent mortality in patients with chronic heart failure. Circulation.

[3] Miró Ò, García Sarasola A, Fuenzalida C (2019). Departments involved during the first episode of acute heart failure and subsequent emergency department revisits and rehospitalisations: an outlook through the NOVICA cohort. Eur J Heart Fail.

[4] Ambrosy AP, Fonarow GC, Butler J (2014). The global health and economic burden of hospitalizations for heart failure: lessons learned from hospitalized heart failure registries. J Am Coll Cardiol.

[5] Barasa A, Schaufelberger M, Lappas G, Swedberg K, Dellborg M, Rosengren A (2014). Heart failure in young adults: 20-year trends in hospitalization, aetiology, and case fatality in Sweden. Eur Heart J.

[6] Takeda A, Martin N, Taylor RS, Taylor SJC (2019) Disease management interventions for heart failure. Cochrane Database Syst Rev 2019(1). 10.1002/14651858.CD002752.PUB4/MEDIA/CDSR/CD002752/IMAGE_N/NCD002752-CMP-003-09.PNG 10.1002/14651858.CD002752.pub4 PMC6492456 30620776

[7] Wan TTH, Terry A, Cobb E, McKee B, Tregerman R, Barbaro SDS (2017) Strategies to modify the risk of heart failure readmission: a systematic review and meta-analysis. 4:233339281770105. 10.1177/2333392817701050 10.1177/2333392817701050 PMC5406120 28462286

[8] van Spall HGC, Rahman T, Mytton O (2017). Comparative effectiveness of transitional care services in patients discharged from the hospital with heart failure: a systematic review and network meta-analysis. Eur J Heart Fail.

[9] Flodgren G, Rachas A, Farmer AJ, Inzitari M, Shepperd S (2015) Interactive telemedicine: effects on professional practice and health care outcomes. Cochrane Database Syst Rev. 2015(9). 10.1002/14651858.CD002098.PUB2/MEDIA/CDSR/CD002098/IMAGE_N/NCD002098-CMP-002-08.PNG 10.1002/14651858.CD002098.pub2 PMC6473731 26343551

[10] Brahmbhatt DH, Cowie MR (2019). Remote management of heart failure: an overview of telemonitoring technologies. Card Fail Rev.

[11] Cleland JGF, Clark RA, Pellicori P, Inglis SC (2020). Caring for people with heart failure and many other medical problems through and beyond the COVID-19 pandemic: the advantages of universal access to home telemonitoring. Eur J Heart Fail.

[12] Achury Saldaña DM, Gonzalez RA, Garcia A, Mariño A, Aponte L, Bohorquez WR (2021). Evaluation of a Mobile Application for Heart Failure Telemonitoring. Comput Inform Nurs.

[13] Inglis SC, Clark RA, Dierckx R, Prieto-Merino D, Cleland JGF (2017). Structured telephone support or non-invasive telemonitoring for patients with heart failure. Heart.

[14] Frederix I, Caiani EG, Dendale P (2019). ESC e-Cardiology Working Group Position Paper: Overcoming challenges in digital health implementation in cardiovascular medicine. Eur J Prev Cardiol.

[15] Allida S, Du H, Xu X, et al (2020) mHealth education interventions in heart failure. Cochrane Database Syst Rev 2020(7). 10.1002/14651858.CD011845.PUB2/MEDIA/CDSR/CD011845/IMAGE_N/NCD011845-CMP-001.05.SVG 10.1002/14651858.CD011845.pub2 PMC7390434 32613635

[16] Wali S, Demers C, Shah H et al (2019) Evaluation of heart failure apps to promote self-care: systematic app Search. JMIR Mhealth Uhealth 7(11):e13173. 10.2196/13173, https://mhealth.jmir.org/2019/11/e13173. Accessed 27 Dec 2021 10.2196/13173 PMC6878098 31710298

[17] Ware P, Ross HJ, Cafazzo JA, Boodoo C, Munnery M, Seto E (2020) Outcomes of a heart failure telemonitoring program implemented as the standard of care in an outpatient heart function clinic: pretest-posttest pragmatic study. J Med Internet Res 22(2):e16538. 10.2196/16538. https://www.jmir.org/2020/2/e16538. Accessed 27 Dec 2021 10.2196/16538 PMC7055875 32027309

[18] Koole MAC, Kauw D, Winter MM (2019). First real-world experience with mobile health telemonitoring in adult patients with congenital heart disease. Neth Hear J.

[19] Foster M (2018). HF app to support self-care among community dwelling adults with HF: a feasibility study. Appl Nurs Res.

[20] Aamodt IT, Lycholip E, Celutkiene J et al (2019) Health care professionals’ perceptions of home telemonitoring in heart failure care: cross-sectional survey. J Med Internet Res 21(2). 10.2196/10362 10.2196/10362 PMC6381407 30724744

[21] Varnfield M, Karunanithi M, Lee CK (2014). Smartphone-based home care model improved use of cardiac rehabilitation in postmyocardial infarction patients: results from a randomised controlled trial. Heart.

[22] Scherr D, Kastner P, Kollmann A et al (2009) Effect of home-based telemonitoring using mobile phone technology on the outcome of heart failure patients after an episode of acute decompensation: randomized controlled trial. J Med Internet Res 11(3):e1252. 10.2196/JMIR.1252. https://www.jmir.org/2009/3/e34. Accessed 27 Dec 2021 10.2196/jmir.1252 PMC2762855 19687005

[23] Neubeck L, Lowres N, Benjamin EJ, Freedman SB, Coorey G, Redfern J (2015). The mobile revolution—using smartphone apps to prevent cardiovascular disease. Nat Rev Cardiol.

[24] Creber RMM, Maurer MS, Reading M, Hiraldo G, Hickey KT, Iribarren S (2016) Review and analysis of existing mobile phone apps to support heart failure symptom monitoring and self-care management using the mobile application rating scale (MARS). JMIR Mhealth Uhealth 4(2):e5882. 10.2196/MHEALTH.5882. https://mhealth.jmir.org/2016/2/e74. Accessed 27 Dec 2021 10.2196/mhealth.5882 PMC4925936 27302310

[25] Koehler F, Koehler K, Deckwart O (2018). Efficacy of telemedical interventional management in patients with heart failure (TIM-HF2): a randomised, controlled, parallel-group, unmasked trial. Lancet.

[26] Cajita MI, Gleason KT, Han HR (2016). A systematic review of mhealth-based heart failure interventions. J Cardiovasc Nurs.

[27] Son YJ, Lee Y, Lee HJ (2020). Effectiveness of mobile phone-based interventions for improving health outcomes in patients with chronic heart failure: a systematic review and meta-analysis. Int J Environ Res Public Health.

[28] Higgins JPT, Thomas J, Chandler J et al (2019) Cochrane handbook for systematic reviews of interventions. Cochrane Handbook for Systematic Reviews of Interventions. 1–694. 10.1002/9781119536604 (Published online January 1)

[29] Liberati A, Altman DG, Tetzlaff J (2009). The PRISMA statement for reporting systematic reviews and meta-analyses of studies that evaluate health care interventions: explanation and elaboration. J Clin Epidemiol.

[30] Bozkurt B, Coats AJS, Tsutsui H (2021). Universal definition and classification of heart failure: a report of the Heart Failure Society of America, Heart Failure Association of the European Society of Cardiology, Japanese Heart Failure Society and Writing Committee of the Universal Definition o. Eur J Heart Fail.

[31] Herdman M, Gudex C, Lloyd A (2011). Development and preliminary testing of the new five-level version of EQ-5D (EQ-5D-5L). Qual Life Res.

[32] Ware JEJ, Sherbourne CD (1992). The MOS 36-item short-form health survey (SF-36). I. Conceptual framework and item selection. Med Care.

[33] Green CP, Porter CB, Bresnahan DR, Spertus JA (2000). Development and evaluation of the Kansas City Cardiomyopathy Questionnaire: a new health status measure for heart failure. J Am Coll Cardiol.

[34] Rector TS (1987) Patient’s self-assessment of their congestive heart failure: II. Content, reli-ability and validity of a new measure-The Minnesota Living with Heart Failure. Heart Fail. https://ci.nii.ac.jp/naid/10005523133/. Accessed 16 June 2022

[35] Wallace BC, Small K, Brodley CE, Lau J, Trikalinos TA (2012) Deploying an interactive machine learning system in an Evidence-based Practice Center: Abstrackr. IHI’12 - Proceedings of the 2nd ACM SIGHIT International Health Informatics Symposium. 819–823. 10.1145/2110363.2110464

[36] New York Heart Association (1994) The criteria committee for the New York heart association. Nomenclature and Criteria for Diagnosis of Diseases of the Heart and Great Vessels. Little, Brown & Co, Boston, MA, USA. (9th ed.):253–255

[37] Schünemann H, Brożek J, Guyatt G, Oxman A (2013) GRADE handbook for grading quality of evidence and strength of recommendations. Updated October 2013. The GRADE Working Group. https://gdt.gradepro.org/app/handbook/handbook.html. Accessed 8 Dec 2021

[38] Vuorinen AL, Leppänen J, Kaijanranta H et al (2014) Use of home telemonitoring to support multidisciplinary care of heart failure patients in Finland: randomized controlled trial. J Med Internet Res 16(12). 10.2196/JMIR.3651 10.2196/jmir.3651 PMC4275484 25498992

[39] Lycholip E, Thon Aamodt I, Lie I (2018). The dynamics of self-care in the course of heart failure management: data from the IN TOUCH study. Patient Prefer Adherence.

[40] Kraai I, de Vries A, Vermeulen K (2016). The value of telemonitoring and ICT-guided disease management in heart failure: Results from the IN TOUCH study. Int J Med Inform.

[41] Hägglund E, Lyngå P, Frie F (2015). Patient-centred home-based management of heart failure. Findings from a randomised clinical trial evaluating a tablet computer for self-care, quality of life and effects on knowledge. Scand Cardiovasc J.

[42] Pekmezaris R, Nouryan CN, Schwartz R (2019). A randomized controlled trial comparing telehealth self-management to standard outpatient management in underserved black and Hispanic patients living with Heart Failure. Telemed J E Health.

[43] Koehler F, Winkler S, Schieber M (2011). Impact of remote telemedical management on mortality and hospitalizations in ambulatory patients with chronic heart failure: The telemedical interventional monitoring in heart failure study. Circulation.

[44] Koehler J, Stengel A, Hofmann T (2021). Telemonitoring in patients with chronic heart failure and moderate depressed symptoms: results of the Telemedical Interventional Monitoring in Heart Failure (TIM-HF) study. Eur J Heart Fail.

[45] Koehler F, Winkler S, Schieber M (2012). Telemedicine in heart failure: pre-specified and exploratory subgroup analyses from the TIM-HF trial. Int J Cardiol.

[46] Galinier M, Roubille F, Berdague P (2020). Telemonitoring versus standard care in heart failure: a randomised multicentre trial. Eur J Heart Fail.

[47] Gjeka R, Patel K, Reddy C, Zetsche N (2021) Patient engagement with digital disease management and readmission rates: the case of congestive heart failure. Health Informatics J 27(3). 10.1177/14604582211030959 10.1177/14604582211030959 34382454

[48] Pedone C, Rossi FF, Cecere A, Costanzo L, Antonelli IR (2015). Efficacy of a physician-led multiparametric telemonitoring system in very old adults with heart failure. J Am Geriatr Soc.

[49] Sahlin D, Rezanezad B, Edvinsson ML, Bachus E, Melander O, Gerward S (2022). Self-care management intervention in heart failure (SMART-HF): a multicenter randomized controlled trial. J Card Fail.

[50] Koehler F, Koehler K, Prescher S (2020). Mortality and morbidity 1 year after stopping a remote patient management intervention: extended follow-up results from the telemedical interventional management in patients with heart failure II (TIM-HF2) randomised trial. Lancet Digital Health.

[51] Dang S, Karanam C, Gómez-Marín O (2017). Outcomes of a mobile phone intervention for heart failure in a minority county hospital population. Telemed J E Health.

[52] Soran OZ, Piña IL, Lamas GA (2008). A randomized clinical trial of the clinical effects of enhanced heart failure monitoring using a computer-based telephonic monitoring system in older minorities and women. J Card Fail.

[53] Clays E, Puddu PE, Luštrek M (2021). Proof-of-concept trial results of the HeartMan mobile personal health system for self-management in congestive heart failure. Sci Rep.

[54] Boyne JJJ, Vrijhoef HJM, Crijns HJGM, de Weerd G, Kragten J, Gorgels APM (2012). Tailored telemonitoring in patients with heart failure: results of a multicentre randomized controlled trial. Eur J Heart Fail.

[55] Gingele AJ, Ramaekers B, Brunner-La Rocca HP (2019). Effects of tailored telemonitoring on functional status and health-related quality of life in patients with heart failure. Neth Hear J.

[56] Kashem A, Droogan MT, Santamore WP, Wald JW, Bove AA (2008). Managing heart failure care using an internet-based telemedicine system. J Card Fail.

[57] Wagenaar KP, Broekhuizen BDL, Jaarsma T (2019). Effectiveness of the European Society of Cardiology/Heart Failure Association website “heartfailurematters.org” and an e-health adjusted care pathway in patients with stable heart failure: results of the “e-Vita HF” randomized controlled trial. Eur J Heart Fail.

[58] Wita M, Orszulak M, Szydło K (2022). The usefulness of telemedicine devices in patients with severe heart failure with an implanted cardiac resynchronization therapy system during two years of observation. Kardiol Pol.

[59] Dorsch MP, Farris KB, Rowell BE, Hummel SL, Koelling TM (2021) The effects of the ManageHF4Life mobile app on patients with chronic heart failure: randomized controlled trial. JMIR Mhealth Uhealth 9(12):e26185. 10.2196/26185, https://mhealth.jmir.org/2021/12/e26185. Accessed 27 Dec 2021 10.2196/26185 PMC8693200 34878990

[60] Dang S, Karanam C, Gómez-Orozco C, Gómez-Marín O (2017). Mobile phone intervention for heart failure in a minority urban county hospital population: usability and patient perspectives. Telemed J E Health.

[61] Melin M, Hägglund E, Ullman B, Persson H, Hagerman I (2018). Effects of a tablet computer on self-care, quality of life, and knowledge: a randomized clinical trial. J Cardiovasc Nurs.

[62] Heidenreich PA, Bozkurt B, Aguilar D (2022). 2022 AHA/ACC/HFSA Guideline for the Management of Heart Failure: A Report of the American College of Cardiology/American Heart Association Joint Committee on Clinical Practice Guidelines. Circulation.

[63] Greene SJ, Ezekowitz JA, Anstrom KJ et al (2022) Medical therapy during hospitalization for heart failure with reduced ejection fraction: the VICTORIA registry. J Card Fail. 10.1016/J.CARDFAIL.2022.02.011 10.1016/j.cardfail.2022.02.011 35301107

[64] Greene SJ, Butler J, Albert NM (2018). Medical therapy for heart failure with reduced ejection fraction: the CHAMP-HF registry. J Am Coll Cardiol.

[65] Bui AL, Fonarow GC (2012). Home monitoring for heart failure management. J Am Coll Cardiol.

[66] Comín-Colet J, Verdú-Rotellar JM, Vela E (2014). Eficacia de un programa integrado hospital-atención primaria para la insuficiencia cardiaca: análisis poblacional sobre 56.742 pacientes. Rev Esp Cardiol.

[67] McDonald M, Virani S, Chan M (2021). CCS/CHFS heart failure guidelines update: defining a new pharmacologic standard of care for heart failure with reduced ejection fraction. Can J Cardiol.

[68] Perez Mv, Mahaffey KW, Hedlin H (2019). Large-scale assessment of a smartwatch to identify atrial fibrillation. N Engl J Med.

[69] Redfors B, Gregson J, Crowley A (2020). The win ratio approach for composite endpoints: practical guidance based on previous experience. Eur Heart J.

