# Supplementary material for: Effectiveness of mobile telemonitoring applications in heart failure patients: systematic review of literature and meta-analysis
Source: Heart Fail Rev. 2023 Jan 18;28(2):431–52. doi: 10.1007/s10741-022-10291-1 (PMC9845822; doi:10.1007/s10741-022-10291-1)
Supplement: Supplementary file 1 — Supplementary file1 (DOCX 189 KB) [file 10741_2022_10291_MOESM1_ESM.docx]

**SUPPLEMENTAL APPENDIX**

Supplemental Methods: **Complete research strategy**

Information will be searched in the following databases: MEDLINE (PUBMED), EMBASE (ELSEVIER) and LILACS (BVSalud). The search terms that will be used for PUBMED are: #1 AND (#2 OR #3 OR #4) AND #5

**PUBMED/Medline**

**#1: ("Heart Failure"[MeSH Terms]) OR ((heart OR cardiac OR myocard*) AND (failure OR insufficien* OR decomp*))**

- "Heart Failure"[MeSH Terms] OR (("heart"[MeSH Terms] OR "heart"[All Fields] OR "hearts"[All Fields] OR "heart s"[All Fields] OR ("cardiacs"[All Fields] OR "heart"[MeSH Terms] OR "heart"[All Fields] OR "cardiac"[All Fields]) OR "myocard*"[All Fields]) AND ("failure"[All Fields] OR "failures"[All Fields] OR "insufficien*"[All Fields] OR "decomp*"[All Fields]))
- Translations
  - heart: "heart"[MeSH Terms] OR "heart"[All Fields] OR "hearts"[All Fields] OR "heart's"[All Fields]
  - cardiac: "cardiacs"[All Fields] OR "heart"[MeSH Terms] OR "heart"[All Fields] OR "cardiac"[All Fields]
  - failure: "failure"[All Fields] OR "failures"[All Fields]

**#2: smartphone OR cellular phone OR cellphone OR iphone OR ipad OR wireless handheld**

- "smartphone"[MeSH Terms] OR "smartphone"[All Fields] OR "smartphones"[All Fields] OR "smartphone s"[All Fields] OR ("cell phone"[MeSH Terms] OR ("cell"[All Fields] AND "phone"[All Fields]) OR "cell phone"[All Fields] OR ("cellular"[All Fields] AND "phone"[All Fields]) OR "cellular phone"[All Fields]) OR ("cell phone"[MeSH Terms] OR ("cell"[All Fields] AND "phone"[All Fields]) OR "cell phone"[All Fields] OR "cellphone"[All Fields] OR "cellphones"[All Fields]) OR ("iphone"[All Fields] OR "iphones"[All Fields]) OR "ipad"[All Fields] OR (("wirel commun mob comput"[Journal] OR "wireless"[All Fields]) AND ("handheld"[All Fields] OR "handhelds"[All Fields]))
- Translations
  - smartphone: "smartphone"[MeSH Terms] OR "smartphone"[All Fields] OR "smartphones"[All Fields] OR "smartphone's"[All Fields]
  - cellular phone: "cell phone"[MeSH Terms] OR ("cell"[All Fields] AND "phone"[All Fields]) OR "cell phone"[All Fields] OR ("cellular"[All Fields] AND "phone"[All Fields]) OR "cellular phone"[All Fields]
  - cellphone: "cell phone"[MeSH Terms] OR ("cell"[All Fields] AND "phone"[All Fields]) OR "cell phone"[All Fields] OR "cellphone"[All Fields] OR "cellphones"[All Fields]
  - iphone: "iphone"[All Fields] OR "iphone's"[All Fields] OR "iphones"[All Fields]
  - wireless: "Wirel Commun Mob Comput"[Journal:__jid101750559] OR "wireless"[All Fields]
  - handheld: "handheld"[All Fields] OR "handhelds"[All Fields]

**#3: mobile applications OR mobile app OR SMS OR short messaging service OR text messaging OR multimedia messag* OR mHealth**

- "mobile applications"[MeSH Terms] OR ("mobile"[All Fields] AND "applications"[All Fields]) OR "mobile applications"[All Fields] OR ("mobile applications"[MeSH Terms] OR ("mobile"[All Fields] AND "applications"[All Fields]) OR "mobile applications"[All Fields] OR ("mobile"[All Fields] AND "app"[All Fields]) OR "mobile app"[All Fields]) OR ("smart mater struct"[Journal] OR "sms"[All Fields]) OR (("short"[All Fields] OR "shorts"[All Fields]) AND ("message"[All Fields] OR "message s"[All Fields] OR "messaged"[All Fields] OR "messages"[All Fields] OR "messaging"[All Fields]) AND ("service"[All Fields] OR "service s"[All Fields] OR "serviced"[All Fields] OR "services"[All Fields] OR "services s"[All Fields] OR "servicing"[All Fields])) OR ("text messaging"[MeSH Terms] OR ("text"[All Fields] AND "messaging"[All Fields]) OR "text messaging"[All Fields]) OR (("multimedia"[MeSH Terms] OR "multimedia"[All Fields] OR "multimedium"[All Fields]) AND "messag*"[All Fields]) OR ("mhealth s"[All Fields] OR "telemedicine"[MeSH Terms] OR "telemedicine"[All Fields] OR "mhealth"[All Fields])
- Translations
  - mobile applications: "mobile applications"[MeSH Terms] OR ("mobile"[All Fields] AND "applications"[All Fields]) OR "mobile applications"[All Fields]
  - mobile app: "mobile applications"[MeSH Terms] OR ("mobile"[All Fields] AND "applications"[All Fields]) OR "mobile applications"[All Fields] OR ("mobile"[All Fields] AND "app"[All Fields]) OR "mobile app"[All Fields]
  - SMS: "Smart Mater Struct"[Journal:__jid9886316] OR "sms"[All Fields]
  - short: "short"[All Fields] OR "shorts"[All Fields]
  - messaging: "message"[All Fields] OR "message's"[All Fields] OR "messaged"[All Fields] OR "messages"[All Fields] OR "messaging"[All Fields]
  - service: "service"[All Fields] OR "service's"[All Fields] OR "serviced"[All Fields] OR "services"[All Fields] OR "services's"[All Fields] OR "servicing"[All Fields]
  - text messaging: "text messaging"[MeSH Terms] OR ("text"[All Fields] AND "messaging"[All Fields]) OR "text messaging"[All Fields]
  - multimedia: "multimedia"[MeSH Terms] OR "multimedia"[All Fields] OR "multimedium"[All Fields]
  - mHealth: "mhealth's"[All Fields] OR "telemedicine"[MeSH Terms] OR "telemedicine"[All Fields] OR "mhealth"[All Fields]

**#4: Telemedicine**

- "telemedicine"[MeSH Terms] OR "telemedicine"[All Fields] OR "telemedicine s"[All Fields]

**#5: 'randomized controlled trial'/exp OR 'controlled clinical trial'/exp OR randomized OR placebo OR randomly OR trial**

- (("randomized controlled trial"[Publication Type] OR "randomized controlled trials as topic"[MeSH Terms] OR "randomized controlled trial"[All Fields] OR "randomised controlled trial"[All Fields]) AND "exp"[All Fields]) OR (("controlled clinical trial"[Publication Type] OR "controlled clinical trials as topic"[MeSH Terms] OR "controlled clinical trial"[All Fields]) AND "exp"[All Fields]) OR ("random allocation"[MeSH Terms] OR ("random"[All Fields] AND "allocation"[All Fields]) OR "random allocation"[All Fields] OR "random"[All Fields] OR "randomization"[All Fields] OR "randomized"[All Fields] OR "randomisation"[All Fields] OR "randomisations"[All Fields] OR "randomise"[All Fields] OR "randomised"[All Fields] OR "randomising"[All Fields] OR "randomizations"[All Fields] OR "randomize"[All Fields] OR "randomizes"[All Fields] OR "randomizing"[All Fields] OR "randomness"[All Fields] OR "randoms"[All Fields]) OR ("placeboes"[All Fields] OR "placebos"[MeSH Terms] OR "placebos"[All Fields] OR "placebo"[All Fields]) OR "randomly"[All Fields] OR ("clinical trials as topic"[MeSH Terms] OR ("clinical"[All Fields] AND "trials"[All Fields] AND "topic"[All Fields]) OR "clinical trials as topic"[All Fields] OR "trial"[All Fields] OR "trial s"[All Fields] OR "trialed"[All Fields] OR "trialing"[All Fields] OR "trials"[All Fields])
- **Translations**
  - **'randomized controlled trial'/:** "randomized controlled trial"[Publication Type] .or. "randomized controlled trials as topic"[MeSH Terms] .or. "randomized controlled trial"[All Fields] .or. "randomised controlled trial"[All Fields]
  - **'controlled clinical trial'/:** "controlled clinical trial"[Publication Type] .or. "controlled clinical trials as topic"[MeSH Terms] .or. "controlled clinical trial"[All Fields]
  - **randomized:** "random allocation"[MeSH Terms] OR ("random"[All Fields] AND "allocation"[All Fields]) OR "random allocation"[All Fields] OR "random"[All Fields] OR "randomization"[All Fields] OR "randomized"[All Fields] OR "randomisation"[All Fields] OR "randomisations"[All Fields] OR "randomise"[All Fields] OR "randomised"[All Fields] OR "randomising"[All Fields] OR "randomizations"[All Fields] OR "randomize"[All Fields] OR "randomizes"[All Fields] OR "randomizing"[All Fields] OR "randomness"[All Fields] OR "randoms"[All Fields]
  - **placebo:** "placeboes"[All Fields] OR "placebos"[MeSH Terms] OR "placebos"[All Fields] OR "placebo"[All Fields]
  - **trial:** "clinical trials as topic"[MeSH Terms] OR ("clinical"[All Fields] AND "trials"[All Fields] AND "topic"[All Fields]) OR "clinical trials as topic"[All Fields] OR "trial"[All Fields] OR "trial's"[All Fields] OR "trialed"[All Fields] OR "trialing"[All Fields] OR "trials"[All Fields]

**Filters:** *English, Spanish, Adult: 19+ years, from 2000/1/1 - 3000/12/12*

**Search Box**: ((("Heart Failure"[MeSH Terms]) OR ((heart OR cardiac OR myocard*) AND (failure OR insufficien* OR decomp*))) AND (smartphone OR cellular phone OR cellphone OR iphone OR ipad OR wireless handheld OR mobile applications OR mobile app OR SMS OR short messaging service OR text messaging OR multimedia messag* OR mHealth OR Telemedicine)) AND ('randomized controlled trial'/exp OR 'controlled clinical trial'/exp OR randomized OR placebo OR randomly OR trial)

**EMBASE**

- **#1: 'heart failure'/exp OR 'heart failure' OR (('heart' OR 'heart'/exp OR heart OR cardiac OR myocard*) AND ('failure' OR 'failure'/exp OR failure OR insufficien* OR decomp*))**
- **#2: 'smartphone' OR 'smartphone'/exp OR smartphone OR (cellular AND phone) OR 'cellphone' OR 'cellphone'/exp OR cellphone OR 'iphone' OR 'iphone'/exp OR iphone OR 'ipad' OR 'ipad'/exp OR ipad OR (wireless AND handheld) OR 'personal digital assistant'/exp OR 'personal digital assistant' OR 'tablet'/exp OR 'tablet'**
- **#3: 'mobile application'/exp OR 'mobile application' OR (mobile AND applications) OR (mobile AND app) OR sms OR (short AND messaging AND service) OR (text AND messaging) OR (('multimedia' OR 'multimedia'/exp OR multimedia) AND messag*) OR 'mhealth' OR 'mhealth'/exp OR mhealth OR 'text messaging'/exp OR 'text messaging'**
- **#4: 'telemedicine' OR 'telemedicine'/exp OR telemedicine**
- **#5: 'randomized controlled trial'/exp OR 'randomized controlled trial' OR 'controlled clinical trial'/exp OR 'controlled clinical trial' OR randomized OR 'placebo'/exp OR placebo OR randomly OR 'trial'/exp OR trial**

**Filters:** AND ([adult]/lim OR [young adult]/lim OR [middle aged]/lim OR [aged]/lim OR [very elderly]/lim) AND ([english]/lim OR [spanish]/lim) AND [2000-2022]/py AND ([embase]/lim OR [preprint]/lim OR [pubmed-not-medline]/lim)

**Search Box:** ('heart failure'/exp OR 'heart failure' OR (('heart' OR 'heart'/exp OR heart OR cardiac OR myocard*) AND ('failure' OR 'failure'/exp OR failure OR insufficien* OR decomp*))) AND ('smartphone' OR 'smartphone'/exp OR smartphone OR (cellular AND phone) OR 'cellphone' OR 'cellphone'/exp OR cellphone OR 'iphone' OR 'iphone'/exp OR iphone OR 'ipad' OR 'ipad'/exp OR ipad OR (wireless AND handheld) OR 'personal digital assistant'/exp OR 'personal digital assistant' OR 'tablet'/exp OR 'tablet' OR 'mobile application'/exp OR 'mobile application' OR (mobile AND applications) OR (mobile AND app) OR sms OR (short AND messaging AND service) OR (text AND messaging) OR (('multimedia' OR 'multimedia'/exp OR multimedia) AND messag*) OR 'mhealth' OR 'mhealth'/exp OR mhealth OR 'text messaging'/exp OR 'text messaging' OR 'telemedicine' OR 'telemedicine'/exp OR telemedicine) AND ('randomized controlled trial'/exp OR 'randomized controlled trial' OR 'controlled clinical trial'/exp OR 'controlled clinical trial' OR randomized OR 'placebo'/exp OR placebo OR randomly OR 'trial'/exp OR trial)

**BVSALUD**

- **#1: (MH: "Heart Failure") OR ((heart OR cardiac OR myocard*) AND (failure OR insufficien* OR decomp*))**
- **#2: smartphone OR "cellular phone" OR cellphone OR iphone OR ipad OR "wireless handheld"**
- **#3: ("mobile applications") OR ("mobile app") OR SMS OR ("short messaging service") OR ("text messaging") OR ("multimedia messag*") OR mHealth**
- **#4: (MH: "Telemedicine") OR telemedicine**
- **#5: (randomized controlled trial) OR (controlled clinical trial) OR randomized OR placebo OR randomly OR trial**

**Filters**: Language: English OR Spanish, Publication years: 2000-2022 Database: exclude MEDLINE.

**Search Box**: ((MH: "Heart Failure") OR ((heart OR cardiac OR myocard*) AND (failure OR insufficien* OR decomp*))) AND (smartphone OR "cellular phone" OR cellphone OR iphone OR ipad OR "wireless handheld" OR ("mobile applications") OR ("mobile app") OR SMS OR ("short messaging service") OR ("text messaging") OR ("multimedia messag*") OR mHealth OR (MH: "Telemedicine") OR telemedicine) AND ((randomized controlled trial) OR (controlled clinical trial) OR randomized OR placebo OR randomly OR trial)

**COCHRANE REVIEWS**

**#1: (heart OR cardiac OR myocard*) AND (failure OR insufficien* OR decomp*)**

**#2: smartphone OR cellular phone OR cellphone OR iphone OR ipad OR wireless handheld**

**#3: mobile applications OR mobile app OR SMS OR short messaging service OR text messaging OR multimedia messag* OR mHealth**

**#4: Telemedicine**

**Filters:** Jan 2000 and Dec 2021.

**Search Box:** ((heart OR cardiac OR myocard*) AND (failure OR insufficien* OR decomp*)) AND (smartphone OR cellular phone OR cellphone OR iphone OR ipad OR wireless handheld OR mobile applications OR mobile app OR SMS OR short messaging service OR text messaging OR multimedia messag* OR mHealth OR Telemedicine)

Supplemental Figure 1. **RoB2**


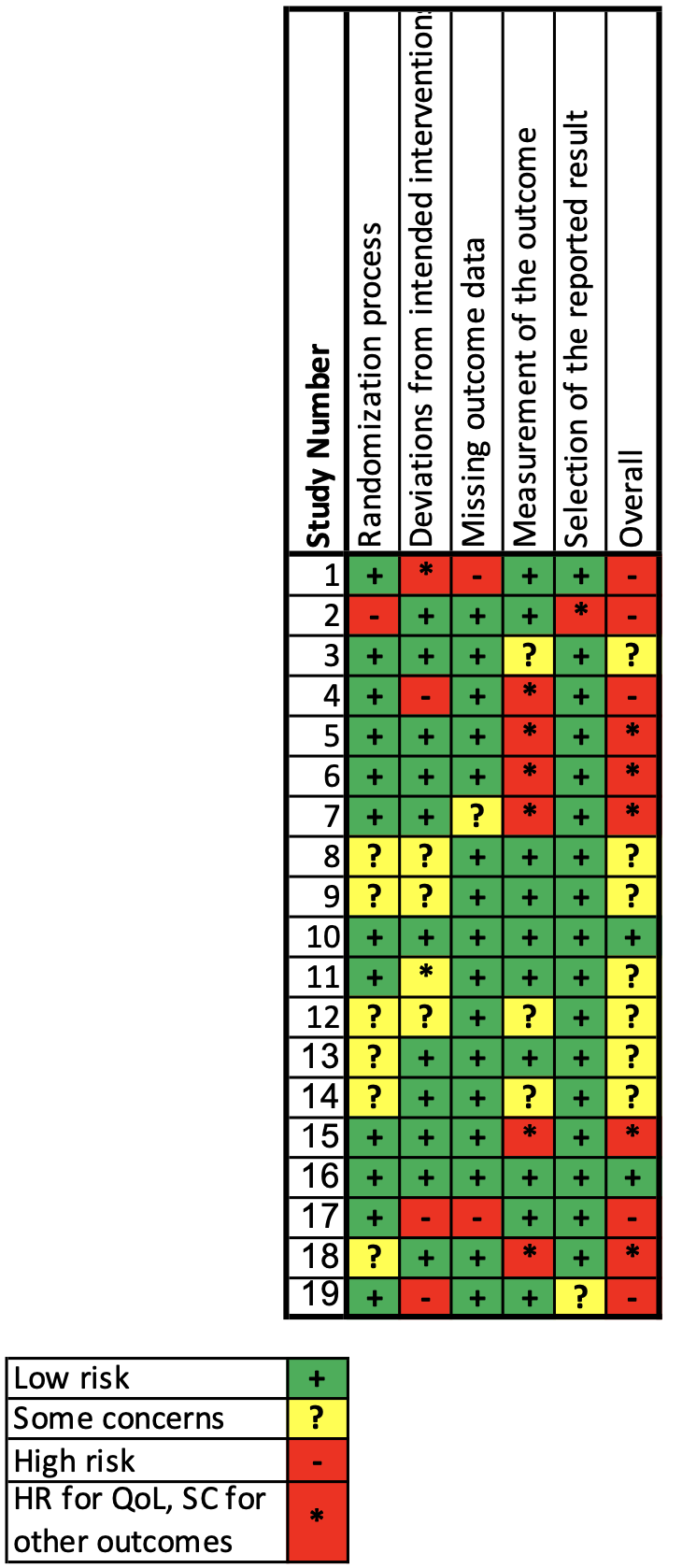


Supplemental Table 1. **GRADE**

**Question:** Mobile application compared to Usual care in Heart failure

**Setting:** for outpatient followup

| **Certainty assessment** | | | | | | | **№ of patients** | | **Effect** | | **Certainty** | **Importance** |
| --- | --- | --- | --- | --- | --- | --- | --- | --- | --- | --- | --- | --- |
| **№ of studies** | **Study design** | **Risk of bias** | **Inconsistency** | **Indirectness** | **Imprecision** | **Other considerations** | **Mobile application** | **Usual care** | **Relative (95% CI)** | **Absolute (95% CI)** |  |  |
| **All cause mortality (follow-up: range 6 months to 26 months)** | | | | | | | | | | | | |
| 10 | randomised trials | serious^a^ | not serious | not serious | not serious | none | 259/2209 (11.7%) | 286/2166 (13.2%) | **RR 0.89** (0.76 to 1.04) | **15 fewer per 1,000** (from 32 fewer to 5 more) | ⨁⨁⨁◯ Moderate | CRITICAL |
| **Cardiovascular mortality (follow-up: range 6 months to 26 months)** | | | | | | | | | | | | |
| 5 | randomised trials | serious^b^ | serious^c^ | not serious | not serious | none | 96/1530 (6.3%) | 124/1523 (8.1%) | **RR 0.78** (0.60 to 1.01) | **18 fewer per 1,000** (from 33 fewer to 1 more) | ⨁⨁◯◯ Low | CRITICAL |
| **All cause hospitalizations (follow-up: range 3 months to 26 months)** | | | | | | | | | | | | |
| 8 | randomised trials | serious^d^ | serious^e^ | not serious | not serious | none | 609/1269 (48.0%) | 600/1214 (49.4%) | **RR 0.93** (0.79 to 1.10) | **35 fewer per 1,000** (from 104 fewer to 49 more) | ⨁⨁◯◯ Low | CRITICAL |
| **Heart failure hospitalizations (follow-up: range 3 months to 26 months)** | | | | | | | | | | | | |
| 12 | randomised trials | serious^f^ | not serious | not serious | not serious | none | 332/1613 (20.6%) | 408/1545 (26.4%) | **RR 0.77** (0.67 to 0.89) | **61 fewer per 1,000** (from 87 fewer to 29 fewer) | ⨁⨁⨁◯ Moderate | CRITICAL |
| **Quality of life (follow-up: range 3 months to 26 months; assessed with: MLHFQ)** | | | | | | | | | | | | |
| 7 | randomised trials | serious^g^ | not serious | not serious | not serious | none | 1167 | 969 | - | **0**  (0 to 0 ) | ⨁⨁⨁◯ Moderate | IMPORTANT |
| **Quality of life (follow-up: range 3 months to 26 months; assessed with: SF-36 )** | | | | | | | | | | | | |
| 2 | randomised trials | very serious^h^ | not serious | not serious | not serious | none | 836 | 811 | - | **0**  (0 to 0 ) | ⨁⨁◯◯ Low | IMPORTANT |
| **Quality of life (follow-up: range 3 months to 26 months; assessed with: EQ-5D)** | | | | | | | | | | | | |
| 1 | randomised trials | not serious | not serious | not serious | not serious | none | 179 | 173 | - | **0**  (0 to 0 ) | ⨁⨁⨁⨁ High | IMPORTANT |
| **Quality of life (follow-up: range 3 months to 26 months; assessed with: KCCQ´s)** | | | | | | | | | | | | |
| 1 | randomised trials | serious^i^ | not serious | not serious | not serious | none | 32 | 40 | - | **0**  (0 to 0 ) | ⨁⨁⨁◯ Moderate | IMPORTANT |

**CI:** confidence interval; **RR:** risk ratio

#### Explanations

a. 2 trials with high risk of bias due to deviations from intended interventions and missing outcome data. 4 trials with some concerns due to randomization process, missing outcome data and measurement of the outcome.

b. 1 trial with high risk of bias due to missing outcome data. 1 trial with some concerns due to randomization process.

c. Wide confidence interval among the trials

d. 1 trial with high risk of bias due to deviations from intended interventions. 4 trials with some concerns due to randomization process, deviations from intended interventions, missing outcome data and measurement of the outcome.

e. 2 trials with different outcome.

f. 2 trials with high risk of bias due to deviations from intended interventions and missing outcome data. 5 trials with some concerns due to randomization process, deviations from intended interventions, missing outcome data and measurement of the outcome.

g. 2 trials with high risk of bias due to measurement of the outcome, 4 trials with some concerns due to randomization process, deviations from intended interventions and measurement of the outcome.

h. 2 trials with high risk of bias due to measurement of the outcome.

i. One trial with high risk of bias due to deviations from intended interventions.
